# Lumi-Guide: An Artificial-Intelligence-Driven Multimodal Framework for Optimizing Personalized Neoadjuvant Therapy Decision-Making in Luminal Breast Cancer

**DOI:** 10.34133/research.1303

**Published:** 2026-05-29

**Authors:** Yanting Liang, Xiaobo Chen, Penghao Lai, Shaoxuan Huang, Minping Hong, Wenjing Jiang, Hongjie Cai, Yujie Ying, Kexin Chen, Kaiqi Hou, Yiying Li, Zhitao Wei, Chinting Wong, Fan Yang, Yuelong Yang, Jie Hou, Zaiyi Liu, Xin Chen, Ying Wang, Changhong Liang, Zhenwei Shi

**Affiliations:** ^1^ Department of Radiology, Guangdong Provincial People’s Hospital, Guangdong Academy of Medical Sciences, Southern Medical University, Guangzhou 510080, China.; ^2^ Guangdong Provincial Key Laboratory of Artificial Intelligence in Medical Image Analysis and Application, Guangzhou 510080, China.; ^3^ Guangdong Cardiovascular Institute, Guangdong Provincial People’s Hospital, Guangdong Academy of Medical Sciences, Guangzhou 510080, China.; ^4^ Department of Radiology, Zhejiang Chinese Medical University Affiliated Jiaxing TCM Hospital, Jiaxing 314033, China.; ^5^ The Second School of Clinical Medicine, Zhejiang Chinese Medical University, Hangzhou 310053, China.; ^6^ Department of Breast Surgery, the First Affiliated Hospital of Zhengzhou University, Zhengzhou 450052, China.; ^7^ Cancer Center, Department of Radiology, Zhejiang Provincial People’s Hospital, Affiliated People’s Hospital, Hangzhou Medical College, Hangzhou 310014, China.; ^8^ Department of Radiology, Guangzhou First People’s Hospital, School of Medicine, South China University of Technology, Guangzhou 510180, China.; ^9^ Department of Medical Ultrasonics, The First Affiliated Hospital of Guangzhou Medical University, Guangzhou 510120, China.

## Abstract

Accurate prediction of pathological complete response (pCR) to neoadjuvant therapy in luminal breast cancer remains challenging, hindering precise treatment decisions. Here, we present the Lumi-Guide system, a novel multimodal framework that integrates deep-learning-based MRI analysis with clinical and genomic data to enable personalized treatment selection. In this multicenter study, we analyzed 1,097 patients with luminal breast cancer from 6 international datasets. We developed a Swin Transformer-based Lumi-I model using 3-plane dynamic contrast-enhanced MRI data and integrated it with clinical factors to construct the Lumi-CI model, which demonstrated robust performance across the validation and 2 external test sets, with areas under the receiver operating characteristic curves of 0.810, 0.819, and 0.864, respectively. Radiogenomic analysis revealed distinct biological characteristics: The Lumi-I high-score group exhibited immunologically active and proliferative microenvironments, while the low-score group showed estrogen-response-driven signaling. To further enhance predictive accuracy, a genomic model (Lumi-G) based on 22 established RNA biomarkers was further developed and integrated with the Lumi-CI model to create a multimodal Lumi-CIG model. We subsequently designed a Lumi-Guide 2-step triage system that prioritizes clinical-imaging information (Lumi-CI) while selectively incorporating genomic data (Lumi-CIG) when beneficial, thus optimizing resource allocation. Critically, by integrating Lumi-Guide system prestratification with response-predictive-subtype-recommended therapies, patients predicted to achieve pCR demonstrated substantially higher actual pCR rates than controls across 3 treatment patterns: 77.8% vs. 61.5% (optimal treatment), 57.6% vs. 24.7% (non-optimal treatment), and 35.3% vs. 4.7% (double-negative response-predictive subtype). This clinically practical and biologically interpretable framework transforms personalized neoadjuvant therapy in luminal breast cancer into a scalable reality.

## Introduction

Breast cancer is the most common malignancy in women worldwide [1], with hormone receptor-positive (HR+)/human epidermal growth factor receptor 2-negative (HER2−) breast cancer, also known as the luminal subtype, accounting for approximately 65% to 70% of all cases [2]. Despite the growing role of neoadjuvant therapy (NAT) in breast cancer management, luminal breast cancer shows limited sensitivity to NAT compared with other breast cancer subtypes, with pathological complete response (pCR) rates of only 7% to 16% [3]. Given that pCR is strongly associated with better long-term survival and quality of life [4], early pCR prediction of NAT in this subtype is clinically essential for (a) identifying patients likely to achieve pCR to avoid premature regimen changes and (b) detecting low-probability responders to prevent ineffective treatment, unnecessary toxicity, and healthcare burden.

To better predict the treatment response in luminal breast cancer, previous studies explore gene expression biomarkers for NAT [5,6]. Wolf et al. [7] established response-predictive subtypes (RPSs) that incorporate tumor biology beyond HR/HER2 status using gene expression data. However, RPS-based optimal treatment allocation yields only modest pCR improvement (35% for luminal subtypes), indicating that current biomarkers insufficiently identify luminal breast cancers benefiting from NAT [7]. More crucially, optimal clinical implementation of these biomarkers remains contentious, particularly regarding their cost-effectiveness and accessibility in resource-limited settings.

Conversely, breast dynamic contrast-enhanced magnetic resonance imaging (DCE-MRI) is routinely performed for cancer staging and therapeutic response assessment, providing comprehensive, noninvasive tumor visualization with dynamic perfusion assessment reflecting tumor biology [8]. Deep-learning approaches have shown promising capability to decode the underlying information contained within medical images noninvasively [9–14].

Despite these advances, several critical limitations persist in current deep-learning research: (a) most models combine all subtypes, ignoring luminal-specific biology; (b) current deep-learning models function as black boxes, limiting clinicians’ understanding of the biological rationale behind predictions; (c) previous studies have mainly used either imaging features or genomic biomarkers alone, whereas the integration of DCE-MRI-based deep learning with RNA-derived biomarkers for cost-effective, multimodal decision systems in this subtype remains unexplored.

Therefore, the aim of this study was to develop a multimodal framework for cost-effective precision treatment decision-making in luminal breast cancer. We created a Swin Transformer-based deep-learning model specific to luminal subtypes and elucidated the biological mechanisms underlying predictions. By integrating imaging features with clinical and genomic signatures, we established a hierarchical decision framework optimizing resource allocation through selective rather than universal genomic testing. We hypothesize this multimodal approach will enhance the treatment-response-prediction accuracy while providing a cost-effective framework for personalized therapeutic strategies in luminal breast cancer.

## Results

### Patient characteristics

**Fig. 1.**
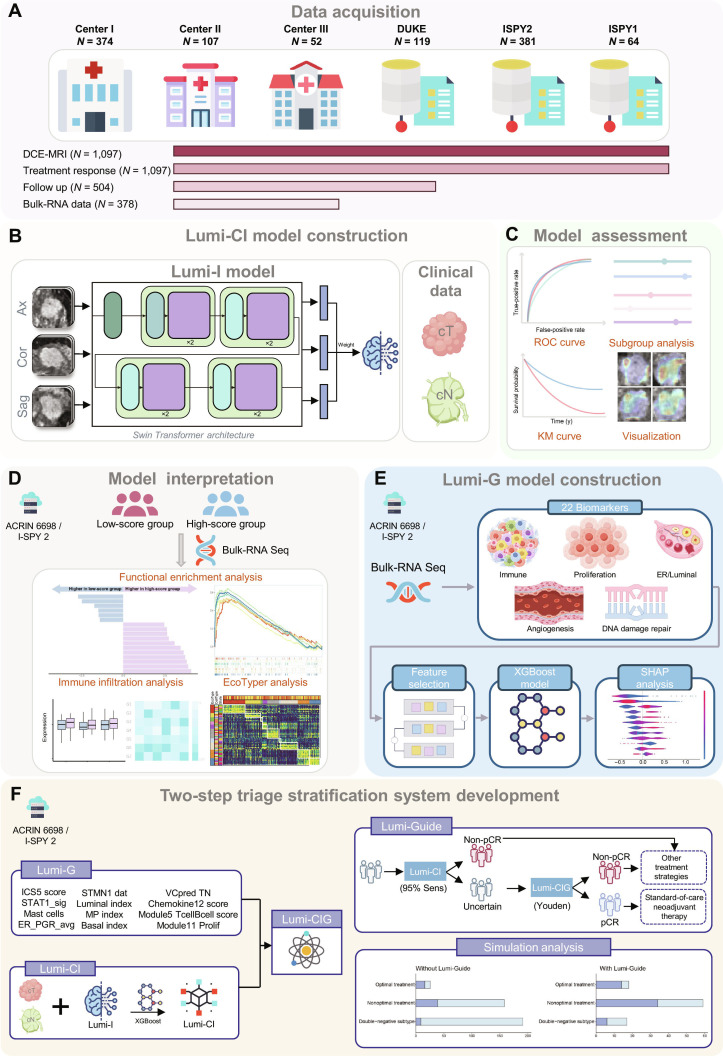
Overview of the study design. (A) Data acquisition. A multicenter dataset of 1,097 patients with luminal breast cancer was collected from 3 hospitals and 3 public datasets. DCE-MRI, dynamic contrast-enhanced magnetic resonance imaging. (B) Lumi-CI model construction. Lumi-I model using Swin Transformer processed multiplanar MRI data, integrated with clinical factors to construct the Lumi-CI model. Sag, sagittal; Cor, coronal; Ax, axial; cT, clinical T stage; cN, clinical N stage. (C) Model assessment. Performance was evaluated using receiver operating characteristic (ROC) analysis, subgroup analysis, Kaplan–Meier (KM) survival analysis, and heatmap visualization. (D) Model interpretation. Functional enrichment, immune infiltration, and EcoTyper analyses provided biological insights. (E) Lumi-G model construction. Genomic biomarkers identified via feature selection and eXtreme Gradient Boosting (XGBoost) algorithm, with Shapley additive explanations (SHAP) interpretability analysis. ER, estrogen receptor. (F) Two-step triage stratification system development. Lumi-CI and Lumi-G models integrated into the Lumi-CIG model. Two-step Lumi-Guide system combined Lumi-CI and Lumi-CIG models and demonstrated superior performance over response-predictive-subtype-based strategies. Some illustrations were generated with www.figdraw.com (License ID: YIUUA54e8c). pCR, pathological complete response; Lumi-C model, Luminal Breast Cancer Clinical model; Lumi-I model, Luminal Breast Cancer Imaging model; Lumi-CI model, Luminal Breast Cancer Clinical-Imaging model; Lumi-G model, Luminal Breast Cancer Genomic model; Lumi-CIG model, Luminal Breast Cancer Clinical-Imaging-Genomic model; Lumi-Guide system, Luminal Breast Cancer Guidance system; ICS5, integrated cytokine score 5; STAT1, signal transducer and activator of transcription; ER, estrogen receptor; PGR, progesterone receptor; STMN1, stathmin 1; MP, MammaPrint; VC, veliparib/carboplatin; TN, triple negative.

This multicenter study included 1,097 patients (median age: 49 years; range: 23 to 85 years) with luminal breast cancer from 3 centers and 3 public datasets. The training, validation, and 2 external test sets (A and B) comprised 612, 262, 107, and 116 patients, respectively, with pCR rates of 10.3% to 16.0%. Patient characteristics are summarized in Table 1, and the study design is shown in Fig. 1.

**Table 1.** Baseline patient clinical characteristics

Characteristic	Training set	Validation set	External test set A	External test set B
Number of patients	612	262	107	116
Age (years) ^a^	48 [42, 55]	47 [40, 55]	51 [42, 59]	51 [45, 61]
Menstrual status				
Premenopausal	332 (54.2)	156 (59.5)	33 (30.8)	25 (21.6)
Postmenopausal	202 (33.0)	76 (29.0)	53 (49.5)	27 (23.3)
Perimenopausal	28 (4.6)	8 (3.1)	21 (19.7)	0 (0.0)
Not available	50 (8.2)	22 (8.4)	0 (0.0)	64 (55.1)
Pre-NAT clinical T stage				
cT1	56 (9.2)	24 (9.2)	13 (12.1)	5 (4.3)
cT2	422 (69.0)	162 (61.8)	70 (65.4)	95 (81.9)
cT3	96 (15.7)	48 (18.3)	14 (13.1)	7 (6.0)
cT4	38 (6.1)	28 (10.7)	10 (9.4)	9 (7.8)
Pre-NAT clinical N stage				
cN0	161 (26.3)	54 (20.6)	19 (17.8)	49 (42.2)
cN1	269 (44.0)	112 (42.7)	71 (66.4)	39 (33.6)
cN2	144 (23.5)	76 (29.0)	9 (8.4)	21 (18.1)
cN3	38 (6.2)	20 (7.7)	8 (7.4)	1 (0.9)
Not available	0 (0.0)	0 (0.0)	0 (0.0)	6 (5.2)
ER status				
Negative	26 (4.2)	7 (2.7)	5 (4.7)	5 (4.3)
Positive	325 (53.1)	135 (51.5)	102 (95.3)	111 (95.7)
Not available	261 (42.7)	120 (45.8)	0 (0.0)	0 (0.0)
PR status				
Negative	55 (9.0)	25 (9.5)	12 (11.2)	15 (12.9)
Positive	296 (48.4)	117 (44.7)	95 (88.8)	99 (85.3)
Not available	261 (42.6)	120 (45.8)	0 (0.0)	2 (1.8)
HER2 status				
Zero	137 (22.4)	57 (21.8)	36 (33.6)	28 (24.1)
Low	335 (54.7)	143 (54.6)	71 (66.4)	24 (20.7)
Not available	140 (22.9)	62 (23.6)	0 (0.0)	64 (55.2)
Ki-67 index				
Low proliferation	43 (7.0)	16 (6.1)	43 (40.2)	15 (12.9)
High proliferation	227 (37.1)	86 (32.8)	64 (59.8)	37 (31.9)
Not available	342 (55.9)	160 (61.1)	0 (0.0)	64 (55.2)
Histological grade				
I	14 (2.3)	8 (3.1)	0 (0.0)	3 (2.6)
II	163 (26.6)	76 (29.0)	0 (0.0)	21 (18.1)
III	137 (22.4)	43 (16.4)	0 (0.0)	11 (9.5)
Not available	298 (48.7)	135 (51.5)	107 (100.0)	81 (69.8)
Treatment response				
pCR	98 (16.0)	42 (16.0)	14 (13.1)	12 (10.3)
Non-pCR	514 (84.0)	220 (84.0)	93 (86.9)	104 (89.7)

Note. Except where indicated, data are numbers of patients with percentages in parentheses. HER2, human epidermal growth factor receptor 2; ER, estrogen receptor; PR, progesterone receptor; NAT, neoadjuvant therapy; pCR, pathologic complete response

^a^ Data are presented as medians with interquartile ranges in brackets.

### Performance of multiplanar Lumi-I and Lumi-CI models

**Fig. 2.**
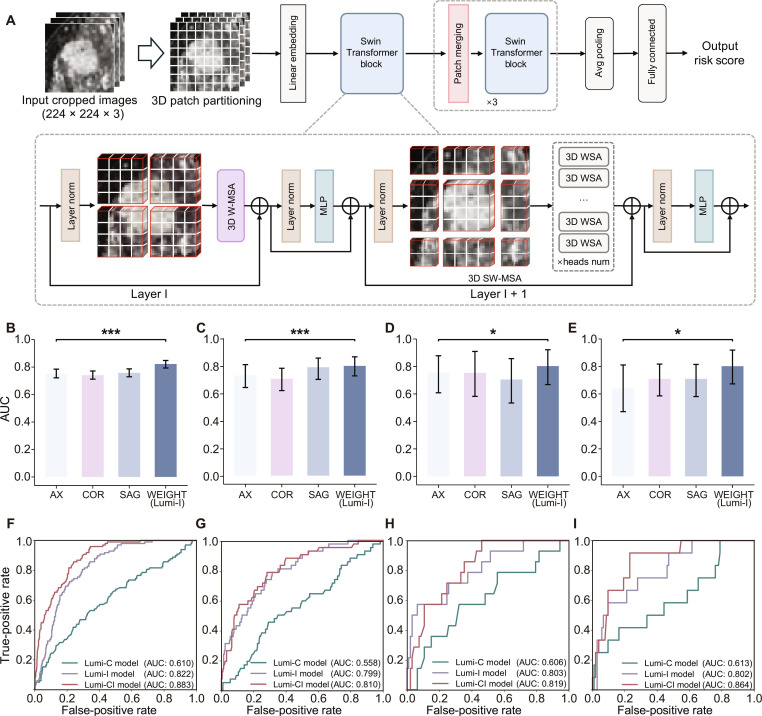
Performance of the models in predicting pCR to neoadjuvant therapy in patients with luminal breast cancer. (A) The Swin Transformer network. (B to E) Area under the receiver operating characteristic (ROC) curves of AX, COR, SAG, and Lumi-I models for the (B) training set, (C) validation set, (D) external test set A, and (E) external test set B, respectively. *P < 0.05; ***P < 0.001. (F to I) ROC curves of Lumi-C, Lumi-I, and Lumi-CI models for the (F) training set, (G) validation set, (H) external test set A, and (I) external test set B, respectively. AX, axial; COR, coronal; SAG, sagittal; AUC, area under the receiver operating characteristic curve; W-MSA, window multi self-attention; SW-MSA, shifted window multi self-attention; WSA, window self-attention; MLP, multilayer perceptron.

The multiplanar Luminal Breast Cancer Imaging (Lumi-I) model (Fig. 2A) outperformed the conventional axial-plane model, achieving areas under the receiver operating characteristic curves (AUCs) of 0.803, 0.799, and 0.802 in the validation test and external test sets A and B, respectively (Fig. 2B to E). To further establish the superiority of the proposed architecture, we compared the Lumi-I model against multiple established baselines, including traditional radiomics models based on 3 machine-learning algorithms and 2 representative convolutional neural networks (VGG16 and ResNet50). The Swin Transformer-based model consistently outperformed these alternative methods across all datasets (Fig. S1). Gradient-weighted class activation mapping (Grad-CAM) visualization revealed that the model focused attention on tumor boundaries and necrotic cores in pCR cases (Fig. S2).

Multivariate analysis identified clinical T (cT) stage and clinical N (cN) stage as independent pCR predictors (*P* < 0.05, Table S1). The Luminal Breast Cancer Clinical (Lumi-C) model showed moderate performance, with a mean AUC of 0.588 ± 0.028 across the validation set and external test sets A and B (Fig. 2F to I). By integrating Lumi-I scores with clinical factors, the Luminal Breast Cancer Clinical-Imaging (Lumi-CI) model achieved superior AUCs of 0.810 (95% confidence interval [CI], 0.741 to 0.880), 0.819 (95% CI, 0.715 to 0.908), and 0.864 (95% CI, 0.751 to 0.947) in the validation set and external test sets A and B (Fig. 2F to I and Table S2), outperforming the Lumi-C model (all *P* < 0.05). Compared with the Lumi-I model, the Lumi-CI model demonstrated higher specificity while maintaining comparable sensitivity (McNemar’s test, *P* < 0.05). Subgroup analyses demonstrated that the Lumi-CI model maintained stable predictive performance across age groups, histological grades, clinical stages, and imaging protocols (Fig. S3). Kaplan–Meier curves indicated that lower Lumi-CI scores were associated with worse disease-free survival (DFS) (Fig. S4A to C).

### Biological mechanisms underlying the MRI-based Lumi-I model

**Fig. 3.**
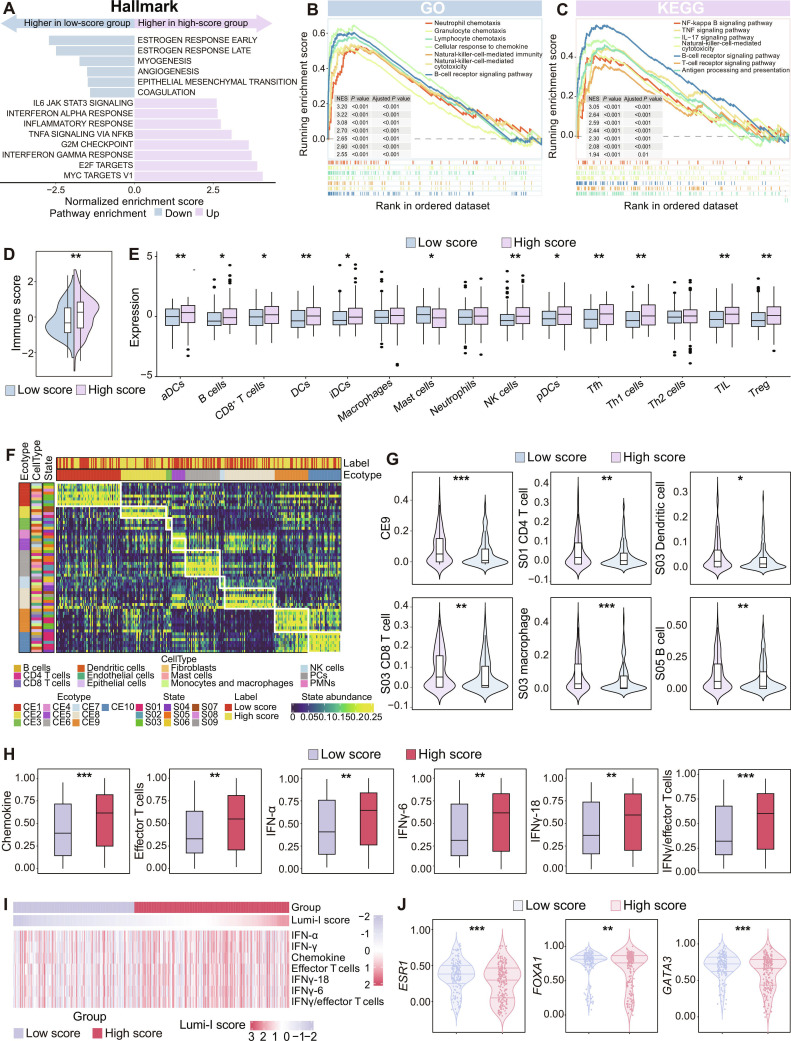
Biological basis of the Lumi-I model. (A) Heatmap showing gene set enrichment analysis of MsigDB Hallmarks gene sets between high-score and low-score groups. (B) GO biological process enrichment analysis illustrating that immune-related pathways are enriched in the high-score group. (C) KEGG pathway enrichment analysis illustrating that immune-related pathways are enriched in the high-score group. (D) Comparison of immune scores between high-score and low-score groups using ESTIMATE algorithm. (E) Immune cell composition analysis showing the fraction of different immune cell types between high-score and low-score groups. (F) The distribution of cell state abundance across 10 carcinoma ecosystems (CEs). (G) Comparison of activated CEs and immune cell state abundances between high- and low-score groups for carcinoma ecotypes 9 (CE9), S01 state CD4^+^ T cells, S03 state dendritic cells, S03 state CD8^+^ T cells, S03 state macrophages, and S05 state B cells, respectively. (H) Validation of immune signatures comparing expression levels between the Lumi-I high-score and low-score groups for chemokine, effector T cells, IFN-α, IFNγ-6, IFNγ-18, and IFNγ/effector-T-cell signature, respectively. (I) The heatmap illustrates the differences in enrichment values of immune-related signatures between high- and low-score groups. (J) Expression comparison of ER-associated genes between high- and low-score groups for *ESR1*, *FOXA1*, and *GATA3*, respectively. GO, Gene Ontology; KEGG, Kyoto Encyclopedia of Genes and Genomes; NES, normalized enrichment score; IFN, interferon; TNF, tumor necrosis factor; IL-17, interleukin 17; aDCs, activated dendritic cells; DCs, dendritic cells; iDCs, immature dendritic cells; NK, natural killer; pDCs, plasmacytoid dendritic cells; Tfh, follicular helper T cells; Th1, type 1 helper T; Th2, type 2 helper T; TIL, tumor-infiltrating lymphocyte; Treg, regulatory T cells; PCs, plasma cells; PMN, polymorphonuclear neutrophil. *P < 0.05, **P < 0.01, ***P < 0.001.

To elucidate the biological basis of the Lumi-I model, we performed comprehensive molecular profiling using MRI and RNA-matched data from the I-SPY2 dataset. To systematically characterize the molecular drivers underlying the model’s predictions, we employed a stepwise analytical approach progressing from pathway-level enrichment to cellular-level immune characterization. Gene set enrichment analysis (GSEA) revealed distinct biological characteristics between Lumi-I high- and low-score groups. Lumi-I high-score tumors demonstrated enrichment in proliferative pathways (E2F targets, G2M checkpoint, and MYC signaling) and immune/inflammatory pathways (interferon [IFN] response and Janus kinase/Signal Transducer and Activator of Transcription signaling) (Fig. 3A to C). Building on these findings, we further characterized the tumor immune microenvironment. Consistent with pathway enrichment, ESTIMATE algorithm demonstrated significantly higher immune scores in the high-score group (*P* < 0.01, Fig. 3D), with elevated infiltration of CD8^+^ T, B, natural killer, and dendritic cells (all *P* < 0.05, Fig. 3E). To more precisely characterize the functional organization of these immune infiltrates, we applied EcoTyper analysis to decipher cell state composition and multicellular communities. This analysis identified significant carcinoma ecotypes 9 (CE9) enrichment with elevated levels of activated immune cells, including effector CD8^+^ T cells (S03), effector CD4^+^ T cells (S01), activated B cells (S05), mature immunogenic dendritic cells (S03), and classically activated M1 macrophages (S03) in the high-score group (all *P* < 0.05; Fig. 3F and G). To validate whether these immune-related findings were linked to immunotherapy response, we assessed the intergroup differences in the expression of established immune signatures and further identified that the high-score group demonstrated marked up-regulation of CD8^+^ T-cell-infiltration signatures, interferon signals (IFN-α, IFNγ-6, and IFNγ-18), effector-T-cell programs, and chemokine activity (Fig. 3H and I).

Conversely, the low-score group showed enrichment in estrogen-response pathways (Fig. 3A) and higher expression of estrogen-response-related genes (Fig. 3J), including *ESR1*, *FOXA1*, and *GATA3*, suggesting that the coordinated activation of this hormone-driven transcriptional network underlies the hormone-dependent phenotype and may contribute to the limited chemotherapy sensitivity observed in this group.

Collectively, these results provide solid biological interpretability for the Lumi-I model, indicating that it captures fundamental tumor biological heterogeneity. Specifically, the model effectively distinguished an immune-proliferative phenotype characterized by activated antitumor immunity and enhanced proliferative dynamics from a hormone-dependent phenotype dominated by estrogen receptor (ER)-driven signaling and diminished immune engagement.

### Added value of genomic biomarker integration

**Fig. 4.**
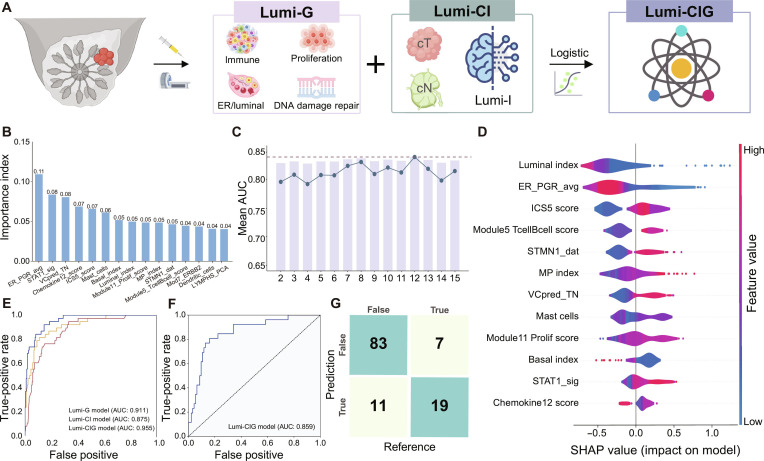
Gene expression biomarker selection and integrated model development. (A) Schematic of the Lumi-CIG model. (B) Ranking of the 15 gene expression biomarkers with variable importance in the radiogenomics discovery set. The sum of the importance scores of each variable is shown at the top of the bars. (C) Relationships between AUC values and the number of features in the radiogenomics discovery set. The *y*-axis shows the AUC, and the *x*-axis shows the corresponding number of features. The red dashed line represents the top mean AUC value. (D) SHAP values of the 12 selected gene expression biomarkers from the Lumi-G model in the radiogenomics discovery set. The bar chart on the upper *x*-axis shows the average SHAP value for each feature as a ranking of importance in descending order. ER_PGR_avg, estrogen receptor–progesterone receptor average; ICS5, integrated cytokine score 5. (E) Comparison of ROC curves among Lumi-G, Lumi-CI, and Lumi-CIG models in the radiogenomics discovery set. (F) ROC curve of the Lumi-CIG model showing its performance in distinguishing pCR from non-pCR patients in the radiogenomics validation set. (G) Confusion matrix showing the prediction distribution for each sample in the radiogenomics validation set. Some illustrations were generated with www.figdraw.com (License ID: YIUUA54e8c).

To investigate whether integrating genomic biomarkers with clinical and imaging features could further enhance pCR prediction performance, we developed and validated an integrated classifier (Fig. 4A) using the radiogenomics discovery and validation sets. Using a random forest with recursive feature elimination method, 12 optimal biomarkers were selected from 22 previously established RNA-based biomarkers (Table S3) associated with NAT response (Fig. 4B and C). The Luminal Breast Cancer Genomic (Lumi-G) model achieved an AUC of 0.911 (95% CI, 0.859 to 0.956) in the radiogenomics discovery set. The Shapley Additive Explanations (SHAP) analysis revealed that luminal index was the most important predictor of pCR, followed by estrogen receptor–progesterone receptor average (ER_PGR_avg) and integrated cytokine score 5 (ICS5) (Fig. 4D).

The multimodal Luminal Breast Cancer Clinical-Imaging-Genomic (Lumi-CIG) model outperformed both the Lumi-CI and Lumi-G models in the radiogenomics discovery set (AUC: 0.955; 95% CI, 0.924 to 0.980, *P* < 0.05; Fig. 4E) and maintained consistent performance in the validation set (AUC: 0.859; 95% CI, 0.778 to 0.931; Fig. 4F and G). The model achieved >80% accuracy in both sets (Table S4).

### Performance and treatment benefit of the Lumi-Guide system

**Fig. 5.**
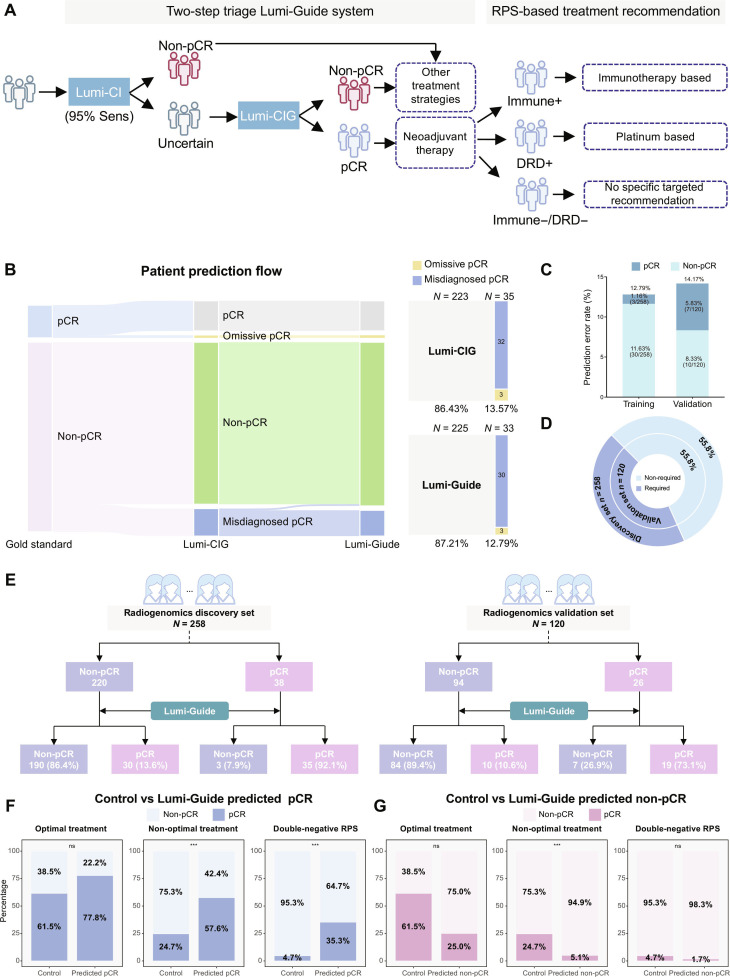
Development and evaluation of the Lumi-Guide stratification system. (A) Schematic of the Lumi-Guide system integrating the response-predictive subtype (RPS)-based treatment recommendation. (B) Left: prediction results flow between Lumi-CIG and Lumi-Guide in the radiogenomics discovery set. Right: proportion matrix of different prediction results. (C) The prediction error rates of the Lumi-Guide system in radiogenomics discovery and validation sets. (D) The proportion of patients in the radiogenomics discovery and validation sets who may not require genetic testing. (E) Recommendation for treatment response according to the Lumi-Guide system in patients with luminal breast cancer in the radiogenomics discovery and validation sets. (F) Pathological complete response rates in Lumi-Guide predicted pCR patients compared to controls in optimal treatment, nonoptimal treatment, and double-negative RPS groups. (G) Pathological complete response rates in Lumi-Guide predicted non-pCR patients compared to controls in optimal treatment, non-optimal treatment, and double-negative RPS groups. ns, not significant; ***P < 0.001.

While the Lumi-CIG model demonstrated superior predictive performance, its reliance on RNA sequencing data presents practical barriers to routine clinical implementation. To address this challenge, we developed the Lumi-Guide (Luminal Breast Cancer Guidance) system, which integrates Lumi-CI and Lumi-CIG models within a 2-step hierarchical triage strategy to optimize the balance between predictive accuracy and resource utilization (Fig. 5A). Using a high-sensitivity threshold (95% sensitivity at Lumi-CI score = 0.238), the system first triages patients into 2 pathways: those unlikely to achieve pCR (ruled out by the Lumi-CI model) and those with uncertain prognosis requiring further genomic evaluation via Lumi-CIG model (Fig. S5). The Lumi-Guide system demonstrated robust performance, with an accuracy of 87.2% in the radiogenomics discovery set and 85.8% in the radiogenomics validation set, respectively (Table S4). Compared with the Lumi-CIG model alone, the Lumi-Guide system maintained comparable pCR identification sensitivity while reducing false-positive predictions (Fig. 5B and Fig. S6), yielding higher specificity in both the radiogenomics discovery (0.864 vs. 0.855) and validation (0.894 vs. 0.883) sets (Table S4). Furthermore, it demonstrated low prediction error rates across both sets (Fig. 5C).

A key advantage of the Lumi-Guide system is its ability to optimize resource allocation. By stratifying patients based on initial Lumi-CI model assessment, the system enabled 55.8% of patients in both sets to avoid genetic testing (Fig. 5D). Clinical analysis showed that 190 (86.4%) and 84 (89.4%) patients could avoid ineffective chemotherapy in discovery and validation sets, with only 3 and 7 patients potentially missing beneficial treatment (Fig. 5E).

We further evaluated whether Lumi-Guide system prestratification could improve treatment outcomes by better matching patients to RPS-recommended therapies (Fig. S7). In the complete radiogenomics set, patients predicted to achieve pCR by the Lumi-Guide system consistently demonstrated higher pCR rates than controls: 77.8% vs. 61.5% (optimal treatment group), 57.6% vs. 24.7% (non-optimal treatment group), and 35.3% vs. 4.7% (double-negative RPS group) (Fig. 5F). Conversely, patients predicted as non-pCR by the Lumi-Guide system showed appropriately lower pCR rates, even when receiving RPS-recommended optimal treatments (Fig. 5G), highlighting the clinical value of Lumi-Guide system prestratification. The results of the discovery and validation sets showed consistent patterns (Fig. S8).

## Discussion

In this multicenter study, we developed the Lumi-CI model, a multiview DCE-MRI deep-learning model integrating clinical information for precision treatment selection in luminal breast cancer. The model achieved robust performance (AUCs of 0.810, 0.819, and 0.864 in the validation set and external test sets A and B, respectively), with biological interpretability through associations with immune activation and proliferation pathways. Further integrating genomic features, we constructed the multimodal Lumi-CIG model, which outperformed both Lumi-CI and Lumi-G models, achieving an AUC of 0.955 (95% CI, 0.924 to 0.980) in the radiogenomics discovery set and 0.859 (95% CI, 0.778 to 0.931) in the validation set. To optimize cost-effectiveness while maintaining clinical accessibility, we developed a Lumi-Guide 2-step triage system that prioritizes imaging and selectively incorporates genomic data when beneficial. Simulation analysis demonstrated that Lumi-Guide system prestratification enhanced RPS-guided treatment effectiveness, providing a systematic framework for personalized neoadjuvant therapy decisions.

Luminal breast cancer exhibits distinct biology with low pCR rates [3], yet most predictive models combine all subtypes, potentially missing subtype-specific features. For instance, Liu et al. [15] and Huang et al. [16] achieved AUCs of 0.71 to 0.82 in validation sets using radiomics across all subtypes. However, this approach may miss the luminal-specific therapeutic response patterns. To address this limitation, our study enrolled 1,097 luminal cases to develop a Swin Transformer-based deep-learning model. Unlike handcrafted radiomics, Swin Transformer architecture effectively captures both fine-grained local features and global contextual information from DCE-MRI images by leveraging its hierarchical feature representation and shifted windowing mechanism. Furthermore, we implemented multiplanar analysis that integrates axial, sagittal, and coronal planes, following radiological practice of multiperspective evaluation. This approach enabled the Lumi-CI model to achieve AUCs of 0.819 and 0.864 in external test sets A and B, respectively.

The clinical model using cT and cN stages exhibited poor performance and low sensitivity, which aligns with findings from previous studies [17,18]. When deep-learning signatures were integrated with clinical factors, performance significantly improved (all *P* < 0.001). This improvement could be attributed to the observation that tumor and nodal stages capture local anatomy, whereas deep-learning signatures assess broader tumor biology, thereby augmenting predictive accuracy.

For clinical adoption, we enhanced model interpretability [19]. Grad-CAM revealed that pCR cases exhibited stronger activation at tumor boundaries and necrotic areas, potentially indicating enhanced immune infiltration at the margins and high proliferative activity, leading to central necrosis. Subsequently, we investigated the biological basis of the Lumi-I model and found that high Lumi-I scores were associated with cell proliferation pathways (MYC and E2F) and immune activation pathways (IFN-γ and IFN-α responses), consistent with previous studies [20–22]. Immune microenvironment analysis further revealed increased immune cells, activated immune cell states, and enrichment of immunoreactive CE9 [23] in the high-score group. Unlike previous studies that reported general associations between models and immune infiltration [24,25], we comprehensively characterized specific immune cell states and multicellular communities. These results indicate that the high-Lumi-I-score group identified tumors with enhanced proliferative dynamics and antitumor immune engagement, potentially underlying their increased sensitivity to therapy. Conversely, tumors with low Lumi-I scores showed enrichment of estrogen-response pathways and overexpression of *ESR1*, *FOXA1*, and *GATA3*, indicating that hormonal signaling contributes to chemotherapy resistance [26–28]. Collectively, these multidimensional validations demonstrate that the Lumi-I model’s predictions are rooted in established luminal breast cancer biology rather than imaging artifacts. By enhancing the biological transparency of the Lumi-I model, our findings confirm its capability to noninvasively identify immune-proliferative versus hormone-dependent phenotypes, providing a reliable tool for precision treatment stratification.

Multimodal machine learning has emerged as a promising strategy for enhanced patient risk stratification [29–32]. However, combining genomic and imaging features for luminal breast cancer treatment-response prediction remains underexplored [33]. In this study, we developed a Lumi-CIG model using matched clinical-imaging genomic data. The Lumi-CIG model outperformed all single-modality approaches, achieving 3.7% and 3.4% higher AUC values than the Lumi-CI and Lumi-G models, respectively. SHAP analysis identified the luminal index, ER_PGR_avg, and ICS5 as key predictors, aligning with previous findings that highlight the importance of hormone signaling and immune infiltration [18].

Although multimodal integration enhances prediction accuracy, comprehensive genomic profiling remains inaccessible in many resource-limited settings [34–36]. To address this barrier, our Lumi-Guide system provides a solution by initially screening patients using readily available clinical-imaging data to identify those unlikely to achieve pCR, while directing remaining patients to undergo genetic testing for more precise prediction using the Lumi-CIG model. This stratified approach enables approximately 55% of patients to avoid genetic testing, while maintaining comparable performance. Patients identified as unlikely to benefit from NAT could be directed toward alternative strategies, such as endocrine therapy or direct surgical resection, avoiding unnecessary toxicity. More critically, the Lumi-Guide system transcends the role of a conventional triage system by functioning as a therapeutic decision-optimizing tool. By integrating Lumi-Guide prestratification with RPS-based treatment recommendations, we transformed a predictive framework into an actionable clinical decision pathway, with pCR rates improving from 61.5%, 24.7%, and 4.7% to 77.8%, 57.6%, and 35.3% across the 3 RPS treatment groups, while predicted non-pCR patients showed lower rates even under optimal therapy (25.0% vs. 61.5%). This selective profiling approach optimizes resource allocation and establishes a scalable, economically feasible framework in luminal breast cancer management.

Our study has several limitations. First, retrospective design has potential selection bias and small genomic sample sizes limited generalizability. Future large prospective cohorts are needed. Second, while DCE-MRI was used as the primary imaging modality, incorporating additional modalities, such as T2-weighted imaging and diffusion-weighted imaging, could provide complementary information and optimize predictive performance. Finally, as a risk stratification tool, our model inevitably generates false-negative results, potentially missing treatment-responsive patients, and multi-timepoint data could reduce misclassification.

In conclusion, the Lumi-CI model, a multiview DCE-MRI deep-learning model, demonstrated robust performance and generalizability by capturing tumor heterogeneity and molecular pathways, including immune-proliferative and hormone-dependent pathways. We further established the value of integrating genomic features with clinical and imaging data and developed a cost-effective 2-step Lumi-Guide system that enhanced RPS-based treatment recommendations providing a practical, resource-optimized solution for personalized NAT decision-making in luminal breast cancer.

## Methods

### Study design and participants

This study was approved by the institutional review boards of 3 hospitals (IRB: KY-Z-2020-680-02), including Guangdong Provincial People’s Hospital (Center I), Zhejiang Provincial People’s Hospital (Center II), and Guangzhou First People’s Hospital (Center III). The requirement for informed consent was waived owing to the retrospective nature of the study.

We consecutively collected 1,097 female patients with luminal breast cancer from 6 datasets, including 3 retrospective and 3 public datasets from The Cancer Imaging Archive (DUKE [37], I-SPY1 [38], and I-SPY2 [39]). The detailed inclusion and exclusion criteria are shown in Fig. S9. The sample size was estimated based on a 7% to 16% pCR rate in luminal breast cancer, as described in Appendix S1.

To maximize imaging protocol diversity and ensure rigorous validation, we combined Center I, I-SPY2, and DUKE datasets into the primary development set. This multisource integration captured a wide array of geographic and protocol variations to foster robust cross-center generalizability. Within this integrated development set, patients were stratified by pCR status and randomly partitioned into training and validation sets in a 7:3 ratio. The remaining datasets were designated as 2 independent external test sets to evaluate cross-site generalizability: Center II formed external test set A (*n* = 107), whereas Center III and the I-SPY1 dataset were merged into external test set B (*n* = 116). Importantly, both external test sets remained strictly isolated throughout the development and hyperparameter-tuning processes to ensure an unbiased evaluation. For the multimodal clinical decision analysis, radiogenomics discovery (*n* = 258) and validation (*n* = 120) sets were derived from the training and validation sets, respectively, by selecting samples with available bulk-RNA sequencing data from the I-SPY2 dataset.

This multicenter study aimed to develop and validate a multimodal framework for predicting pCR to neoadjuvant therapy in luminal breast cancer and to establish a clinically implementable system for treatment optimization. The study was designed with the following components: (a) development of a deep-learning-based imaging model (Lumi-I) using multiplanar dynamic contrast-enhanced MRI data, integrated with clinical factors to construct Lumi-CI model; (b) radiogenomic analysis to elucidate biological underpinnings of imaging predictions; (c) development of a genomic model (Lumi-G) based on established RNA biomarkers and integration with the Lumi-CI model to create a multimodal Lumi-CIG model; (d) design and validation of a Lumi-Guide 2-step triage system for resource-efficient treatment selection; and (e) evaluation of treatment benefit through stratified analysis integrating Lumi-Guide system predictions with RPS-directed therapy recommendations.

### Neoadjuvant therapy regimens and clinicopathological data acquisition

All patients received at least 4 cycles of NAT treatment. The regimens involved taxane-based, alkylator-based, and anthracycline-based chemotherapy alone, or in combination with immunotherapy or targeted therapy. pCR was defined as no residual invasive carcinoma (ypT0/is ypN0). Clinicopathological data (age, menstrual status, cT stage, cN stage, ER, progesterone receptor, HER2, and Ki-67 index) were retrospectively collected. Detailed information regarding the histopathological data is provided in Appendix S2. DFS was defined as the time from the date of surgery to the first event of disease recurrence, metastasis, death from any cause, or date of the most recent follow-up. The minimum follow-up period required to ascertain DFS was 12 months.

### Image acquisition and preprocessing

All MRI scans were performed before NAT by using a 1.5- or 3.0-T scanner. Images from the peak tumor enhancement phase on DCE-MRI were used for further analyses. The details of scanning parameters are provided in Table S5.

An in-house semi-automated segmentation tool, SwinHR [40], was used to improve the efficiency and accuracy of tumor delineation. Subsequently, the preliminary segmented volumes of interest were further revised by a radiologist with >6 years of experience. To reduce intercenter heterogeneity and standardize model input, all images underwent a unified preprocessing pipeline before model development, including N4 bias field correction, isotropic resampling to 1 × 1 × 1 mm^3^, imaging cropping based on the tumor volume of interest, and intensity normalization. The detailed preprocessing procedures are provided in Appendix S3.

### Development of the multiplanar Lumi-I model

A Swin Transformer-based multiplanar deep-learning model was developed to predict NAT response in luminal breast cancer. An ImageNet-pretrained Swin-Tiny architecture was used as the backbone. Three plane-specific models were independently trained on images from the axial, coronal, and sagittal planes, respectively. Each model was optimized using the binary cross-entropy loss function in conjunction with the AdamW optimizer, and the 3 plane-specific prediction scores were subsequently integrated by logistic regression to construct the final Luminal Breast Cancer Imaging (Lumi-I) model. Detailed network configuration and training hyperparameters are described in Appendix S4. The output probability of pCR was defined as the Lumi-I score. Patients were stratified into high- and low-score groups based on an optimal cutoff value determined by the maximum Youden index in the training set. To identify and visualize the image regions that contribute to the model’s prediction, Grad-CAM was employed, with the detailed visualization procedures described in Appendix S5.

To benchmark the performance of the Lumi-I model, comparisons were conducted against traditional radiomics models based on 3 machine-learning algorithms (decision tree, support vector machine, and eXtreme Gradient Boosting [XGBoost]) as well as alternative deep-learning architectures (VGG16 and ResNet50). All models were trained and evaluated on the same dataset under identical preprocessing pipelines and experimental protocols. Detailed descriptions of the baseline methods are provided in Appendix S6.

### Development of the Lumi-C and Lumi-CI models

Clinical factors, including age, cT stage, and cN stage, were incorporated into both univariate and multivariate logistic regression analyses to identify independent predictors and construct the Lumi-C model. Subsequently, the Lumi-CI model was constructed based on the XGBoost algorithm by integrating these significant clinical predictors with the Lumi-I score. The optimal hyperparameters were optimized through a grid search and 5-fold cross-validation (Table S6).

To evaluate the robustness and generalizability of the Lumi-CI model, subgroup analyses were performed according to age, histological grade, clinical T stage, clinical N stage, MRI field strength, and scanner manufacturer. Furthermore, to assess the prognostic performance of the Lumi-CI model, patients with available DFS information were stratified into high-risk and low-risk groups based on the optimal cutoff calculated using the minimum log-rank *P*-value method in Center I. Prognostic differences were assessed using Kaplan–Meier analysis and log-rank tests.

### Biological interpretability analysis of the Lumi-I model

To elucidate and validate the biological basis of the Lumi-I model and enhance its interpretability, we performed pathway enrichment analysis using MRI and RNA-matched data from the I-SPY2 dataset. GSEA was employed to investigate biological function and pathway enrichment differences between high- and low-score groups based on Gene Ontology, Kyoto Encyclopedia of Genes and Genomes, and Hallmark gene sets. Significant pathways were selected using a false discovery rate of <0.05 and |normalized enrichment score| > 1.

We further profiled the tumor immune microenvironment using a multifaceted computational approach: ESTIMATE algorithm was applied to calculate the overall immune scores; the single sample GSEA algorithm was used to quantify the infiltration levels of 15 immune cell types [41]; gene set variation analysis was employed to evaluate IFN signaling activity [42], including IFNγ-6 [43] and IFNγ-18 [44] signature; and EcoTyper [23] was utilized to systematically identify and quantify the relative abundances of different cell states and carcinoma ecotypes, thereby characterizing tumor-microenvironment composition associated with treatment-response predictions.

### Genomic biomarker integration and multimodal model development

Based on 22 multidimensional RNA-based biomarkers [7] associated with NAT identified in previous studies (Table S3), we constructed a Lumi-G model using a 2-step feature selection strategy within the radiogenomics discovery set. Initially, random forest with recursive feature elimination was applied to assess the feature importance of each biomarker. The optimal feature subset was then selected based on the highest mean AUC value derived from 5-fold cross-validation among the top 15 ranked features. The final Lumi-G model was constructed using the XGBoost algorithm with the optimized hyperparameters provided in Table S6. The SHAP analysis was used to quantify the contribution of each feature. Subsequently, a multimodal Lumi-CIG model was developed using logistic regression by integrating the output probabilities of the Lumi-CI and Lumi-G models to enhance predictive accuracy.

### Lumi-Guide system development and implementation strategy

To rationalize resource allocation while maintaining predictive accuracy, we implemented a 2-step hierarchical triage strategy named Lumi-Guide system combining Lumi-CI and Lumi-CIG models sequentially. In the first step, all patients were screened using the Lumi-CI model with a high-sensitivity cutoff (95% sensitivity) to identify and rule out patients unlikely to achieve pCR. In the second step, the remaining patients with uncertain status underwent RNA profiling and were further evaluated using the comprehensive Lumi-CIG model to be definitively classified as predicted pCR or non-pCR according to Youden’s index-optimized cutoff. This hierarchical approach ensures that the more resource-intensive RNA-based testing is reserved only for the subset of patients in whom the initial clinical-imaging assessment yields uncertain results, thereby enhancing cost-effectiveness without compromising diagnostic accuracy.

### Assessment of treatment benefit with Lumi-Guide system stratification

To evaluate whether Lumi-Guide system stratification could optimize treatment selection and improve patient outcomes, we conducted a stratified comparative analysis using the RPS framework as the treatment recommendation.

RPS incorporates comprehensive tumor biological features by defining molecular subtypes based on immune phenotype and DNA-repair-deficiency phenotype (detailed methods in Appendix S5). A recommended therapy based on RPS was assigned as follows [7]: immune-positive type (recommendation of immunotherapy-based regimens), DNA-repair-deficiency-positive type (recommendation of platinum-based chemotherapy regimens), and double-negative RPS type (no specific targeted recommendation).

We compared pCR rates under 2 scenarios: (a) control scenario: all patients were stratified into 3 groups based on concordance between RPS-recommended and actual received treatments—optimal treatment group (received RPS-recommended therapy), nonoptimal treatment group (did not receive matched therapy), and double-negative RPS group (no specific recommendation available); and (b) Lumi-Guide system intervention scenario: patients were first stratified by the Lumi-Guide system into predicted non-pCR and pCR subgroups. Within each Lumi-Guide-predicted subgroup, patients were further classified according to the same RPS concordance criteria. pCR rates between scenarios were compared using the chi-squared test to assess the added benefit of Lumi-Guide system stratification.

### Statistical analysis

Continuous variables were compared using the independent *t* test or Mann–Whitney *U* test, and categorical variables were compared using Fisher’s exact test or chi-squared test, as appropriate. Model performance was evaluated using AUC, accuracy, sensitivity, and specificity. The DeLong test compared AUC differences, with 95% CIs calculated from 1,000 bootstrap replicates [45]. The optimal cutoff values were determined using the maximum Youden index, except when otherwise noted. Two-sided *P* <0.05 was considered significant.

## Data Availability

The raw in-house data are protected and not available because of data privacy laws, while supporting the findings, including the imaging data, can be available under restricted access for noncommercial and academic purposes only. Duke Breast Cancer MRI data are available at TCIA (https://www.cancerimagingarchive.net/collection/duke-breast-cancer-mri/). The I-SPY2 Breast Cancer MRI datasets are available at the TCIA (https://www.cancerimagingarchive.net/collection/ispy2/). The I-SPY1 Breast Cancer MRI datasets are available at TCIA (https://www.cancerimagingarchive.net/collection/ispy1/). The source codes used in this study are open source and are available at https://github.com/Englishday/Lumi-I_model. The transcriptomic data analyzed in this study were obtained from Gene Expression Omnibus (GEO) at GSE194040 (https://www.ncbi.nlm.nih.gov/geo/query/acc.cgi?acc=GSE194040).

## Supplementary Materials

Supplementary 1 Supplementary Appendixes 1 to 7 Figs. S1 to S9 Tables S1 to S7
