# Supplementary material for: Lumi-Guide: An Artificial-Intelligence-Driven Multimodal Framework for Optimizing Personalized Neoadjuvant Therapy Decision-Making in Luminal Breast Cancer
Source: Research (Wash D C). 2026 May 29;9:1303. doi: 10.34133/research.1303 (PMC13220084; doi:10.34133/research.1303)
Supplement: Supplementary 1 — Supplementary Appendixes 1 to 7 Figs. S1 to S9 Tables S1 to S7 [file research.1303.f1.zip › Supplement-CleanVersion.docx]

**Supplementary information for**

**Lumi-Guide: An AI-Driven Multimodal Framework for Optimizing Personalized Neoadjuvant Therapy Decision-Making in Luminal Breast Cancer**

Supplementary Appendix 1: Sample size evaluation

Supplementary Appendix 2: Definitions of molecular receptor status

Supplementary Appendix 3: Segmentation reliability assessment and image preprocessing

Supplementary Appendix 4: The Lumi-I model design and implementation details

Supplementary Appendix 5: Model visualization and interpretability analysis

Supplementary Appendix 6. Baseline model development for comparative analysis

Supplementary Appendix 7: Implementation of previously published response-predictive subtypes schemas

**Supplementary Appendix 1. Sample size evaluation**

The sample size evaluation of this retrospective study was determined using the PASS2021 software. For the luminal breast cancer treatment response prediction task, an original hypothetical area under the curve (AUC0) value of 0.70, an expected AUC1 value of 0.85, an alpha level of 0.05, a power of (1-β = 0.85), a percent value of 10% (according to the rate of pCR after NAT around 7%-16%) and a 20% dropout rate were assumed. The results suggested that we require a minimum sample size of 330 participants. In this study, 1,097 patients were enrolled for the imaging model to predict pCR, and 378 patients were included for the clinical-imaging-genomic model, both of which were sufficient for the analysis.

**Supplementary Appendix 2. Definitions of molecular receptor status**

Nuclear staining of ER/PR by immunohistochemistry (IHC) with ≥1% positive tumor cells was defined as ER/PR positive, while staining with <1% positive tumor cells was defined as ER/PR negative. HER2 status was determined via immunohistochemistry, with 0 or 1+ considered negative, 3+ considered positive, and 2+ requiring further fluorescence in situ hybridization. The Ki-67 index was considered high if it was ≥20%.

**Supplementary Appendix 3. Segmentation reliability assessment and image preprocessing**

The preliminary segmented volumes of interests (VOIs) were further revised with review by radiologist (R1) with > 6 years of experience. Tumor VOIs were defined to encompass all visible tumor parenchyma, including enhancing solid components and intratumoral necrotic areas, while peritumoral edema and normal fibroglandular tissue were explicitly excluded. To assess inter-observer reliability, 100 cases were randomly selected, and another experienced radiologist (R2) independently re-revised these 100 cases. The Dice similarity coefficient was used to quantify agreement between R1 and R2. A Dice score of 0.875 was achieved, indicating strong inter-observer segmentation consistency. For intra-observer reliability, radiologist R1 repeated the segmentation of the same 100 randomly selected cases after a 1-month interval to minimize recall bias. Agreement between the two rounds of annotations was again evaluated using the Dice similarity coefficient, and a Dice score of 0.898 was obtained, confirming robust intra-observer reproducibility.

To ensure consistency across multicenter data while accommodating protocol variations, a comprehensive standardized preprocessing pipeline was implemented using identical software tools and parameters across all centers. First, N4 bias field correction was applied to all images to remove low-frequency intensity non-uniformities arising from magnetic field inhomogeneities. Second, all images were resampled to an isotropic spatial resolution of 1×1×1 mm³ in the axial, coronal, and sagittal planes to harmonize differences in slice thickness and spatial resolution across centers. Gray-scale images were resampled using third-order B-spline interpolation, whereas binary masks were resampled using nearest-neighbor interpolation to preserve lesion boundaries. Third, the axial slice containing the largest tumor cross-sectional area S_max_ and its two adjacent slices S_max±1_ were identified. For each slice, a minimal square bounding box enclosing the tumor volume of interest was generated, with an additional 10-pixel margin to retain the tumor-parenchyma interface. The cropped region was then resized to 224×224 pixels using bilinear interpolation. Corresponding coronal and sagittal images were cropped and resized using the same procedure. Finally, intensity values were normalized using the mean and standard deviation calculated from the training set for each anatomical plane. For downstream model development, the three consecutive tumor-centered slices in each plane were stacked as a three-channel image to construct a 2.5D input of size 224×224×3. This preprocessing strategy standardized model input while preserving peritumoral contextual information.

**Supplementary Appendix 4. The Lumi-I model design and implementation details**

We trained the Luminal Breast Cancer Imaging (Lumi-I) model to predict pathological complete response in luminal breast cancer patients. The Lumi-I model was developed based on a Swin Transformer architecture with a classification head sequentially composed of a global average pooling layer and a fully connected linear layer. The Swin Transformer partitions the input image into non-overlapping local windows and performs self-attention computation within each window, enabling the model to efficiently capture local tumor structures and texture patterns. By alternating between regular window-based multi-head self-attention (W-MSA) and shifted-window multi-head self-attention (SW-MSA) across consecutive Transformer blocks, the architecture facilitates cross-window information exchange and hierarchical feature aggregation across multiple scales. Within each window of $M\times M$ patches, self-attention is computed as:

$$Attention(Q,K,V)=Softmax(\frac{QK^{T}}{\sqrt{d_{k}}}+B)V$$

where $Q,K,V\in\mathbb{R}^{M^{2}\times d}$ are the query, key, and value matrices, $d_{k}$ is the per-head feature dimension, and $B\in\mathbb{R}^{\left( 2M-1)\times(2M-1 \right)}$ is a learnable relative position bias matrix.

The ImageNet-pretrained Swin-Tiny variant (swin_tiny_patch4_window7_224) was adopted as the backbone. The input images were fed into the model with a patch size of 4×4 and a window size of 7×7. The embedding dimension at the first stage was 96, with stage depths of [2, 2, 6, 2] and numbers of attention heads of [3, 6, 12, 24] across the four hierarchical stages. The final feature map was reduced to a 1×768 vector via global average pooling, which was then passed through a linear layer to output a scalar pCR probability.

Three plane-specific models were independently fine-tuned on axial, coronal reformatted, and sagittal reformatted images, respectively, using the pretrained weights as initialization. For each plane, the model input was a 2.5D representation of size 224×224×3 formed by stacking the tumor-maximal slice and its two adjacent slices as three channels. To increase the training data and avoid overfitting of the network, data augmentation was performed during fine-tuning, which included random rotation, random horizontal and vertical flipping, and color jitter with brightness and contrast perturbation. Model optimization minimized the binary cross-entropy loss:

$$\mathcal{L}_{\mathrm{CE}}=-\frac{1}{N}\sum_{i=1}^{N} [y_{i}log{\overset{^}{p}}_{i}+(1-y_{i})log(1-{\overset{^}{p}}_{i})]$$

where $N$ is the batch size, $y_{i}\in\{0,1\}$is the ground-truth pCR label, and $\hat{p}_{i}$ is the predicted pCR probability for the $i$-th sample. The AdamW optimizer was employed with an initial learning rate of 4×10^-6^, weight decay of 0.0001, and batch size of 132. Training proceeded for 80 epochs with a cosine annealing learning rate schedule, in which the learning rate was decayed from 8×10^-7^ to a minimum of 1×10^-9^ over a cycle period of 40 epochs.

For risk prediction, each planar model generated a plane-specific risk score (AXscore, CORscore, SAGscore) based on predictions from S_max_ and its two adjacent slices (S_max±1_). Subsequently, a multi-planar ensemble model was developed by applying logistic regression with coefficient weighting on the upsampled training dataset to integrate information from all three anatomical orientations. The final weighted prediction score was calculated as follows:

**Weight Score** = 1.896*AXscore + 1.538*CORscore + 3.892*SAGscore - 2.178

The output of this fusion model was defined as the final Lumi-I score and was used for subsequent construction of the Luminal Breast Cancer Clinical-Imaging (Lumi-CI) model. All experiments were conducted using Python (version 3.8.19) with PyTorch on a workstation equipped with an Intel i9-14900K CPU and an NVIDIA GeForce RTX 4090 GPU.

**Supplementary Appendix 5. Model visualization and interpretability analysis**

To enhance model interpretability and localize the image regions most influential to the Lumi-I model’s pCR predictions, Gradient-weighted Class Activation Mapping (Grad-CAM) was employed. This method localizes important regions by visualizing class-specific gradient information that determines the final classification prediction.

In Grad-CAM, the neuron importance weight $\alpha_{k}^{c}$or a specific class c is calculated as:

$$\alpha_{k}^{c}=\frac{1}{Z}\sum_{i} \sum_{j} \frac{\partial y^{c}}{\partial A_{\mathrm{ij}}^{k}}$$

where $y^{c}$ denotes the gradient score for class c (pCR or non-pCR) before the softmax layer, $A^{k}$represents the feature map activation of the kth layer in the classification network, and Z is a normalization constant. After computing the neuron importance weights for each feature map, we generate a heat map indicating the significant regions related to class c by performing a weighted linear combination of the feature maps, followed with a ReLU activation function:

$$L_{Grad-CAM}^{c}=ReLU \left( \sum_{k} \alpha_{k}^{c}A^{k} \right)$$

**Supplementary Appendix 6. Baseline model development for comparative analysis**

To benchmark the Lumi-I model, three baseline models were developed and evaluated under identical preprocessing pipelines and experimental protocols.

Traditional Radiomics Model: Radiomic features were extracted from VOIs on DCE-MRI using PyRadiomics (version 3.9.6), yielding 944 features per patient (including original, square, and wavelet features), followed by Z-score normalization. Feature selection was performed stepwise: features associated with pCR (*P* < 0.05) were first identified using the Mann-Whitney U test, and highly correlated features were removed based on Spearman’s correlation analysis (|r| ≥ 0.6). The remaining features were further selected using least absolute shrinkage and selection operator with elastic net regularization, with the optimal λ determined via 5-fold cross-validation, and features with non-zero coefficients were retained. The final features set were detailed in Supplementary Table S7. Based on these selected features, three machine learning models (decision tree, support vector machine and eXtreme Gradient Boosting) were constructed, with hyperparameters optimized using grid search and 5-fold cross-validation.

VGG16 and ResNet50 Models: To ensure a fair comparison, both VGG16 and ResNet50 models adopted the same multi-planar input strategy and training framework as the Lumi-I model. Three plane-specific models were independently trained on AX, COR, and SAG images, respectively, and the plane-specific prediction scores were subsequently integrated via logistic regression to generate the final prediction. For the VGG16 model, an ImageNet-pretrained VGG16 served as the backbone, with the final fully connected layer replaced by a binary classification head. For the ResNet50 model, an ImageNet-pretrained ResNet50 served as the backbone, with the final fully connected layer replaced by a binary classification head. Both models were optimized using the binary cross-entropy loss function with the AdamW optimizer, and all training hyperparameters were kept identical to those of the Lumi-I model, as detailed in Supplementary Appendix S4.

**Supplementary Appendix 7. Implementation of previously published response-predictive subtypes schemas**

Based on RNA sequencing data in the I-SPY2 dataset, we evaluated response-predictive subtypes (RPS) based on a previous study: the Immune phenotype and DNA repair deficiency (DRD) phenotype. For immune phenotype classification in HR+HER2- patients, we applied a previously validated dual-marker approach combining B cell infiltration (sensitivity marker) and mast cell presence (resistance marker), using established thresholds of 0.1495 and 1.17 respectively to define Immune-high (B cells ≥ 0.1495 and Mast cells < 1.17) versus Immune-low groups (B cells < 0.1495 or Mast cells ≥ 1.17). DRD status was determined using BP-Basal classification, classifying BP-Basal tumors as DRD+ and BP-Luminal as DRD-.


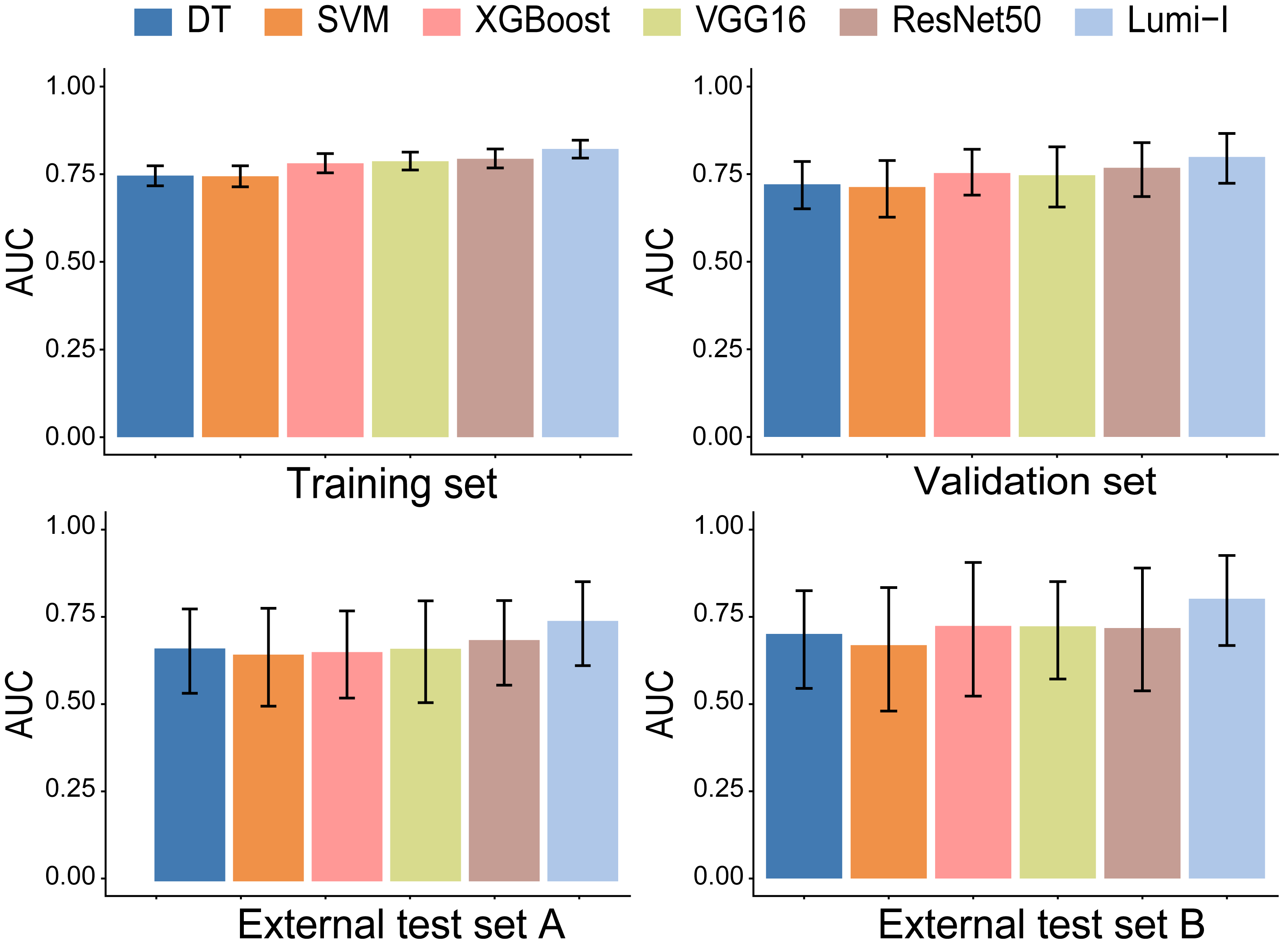


**Supplementary Fig. S1. Performance comparison of the Lumi-I model and baseline methods across different datasets.** Abbreviations: DT, decision tree; SVM, support vector machine; XGBoost, eXtreme Gradient Boosting; Lumi-I model, Luminal Breast Cancer Imaging model; AUC, area under the curve.


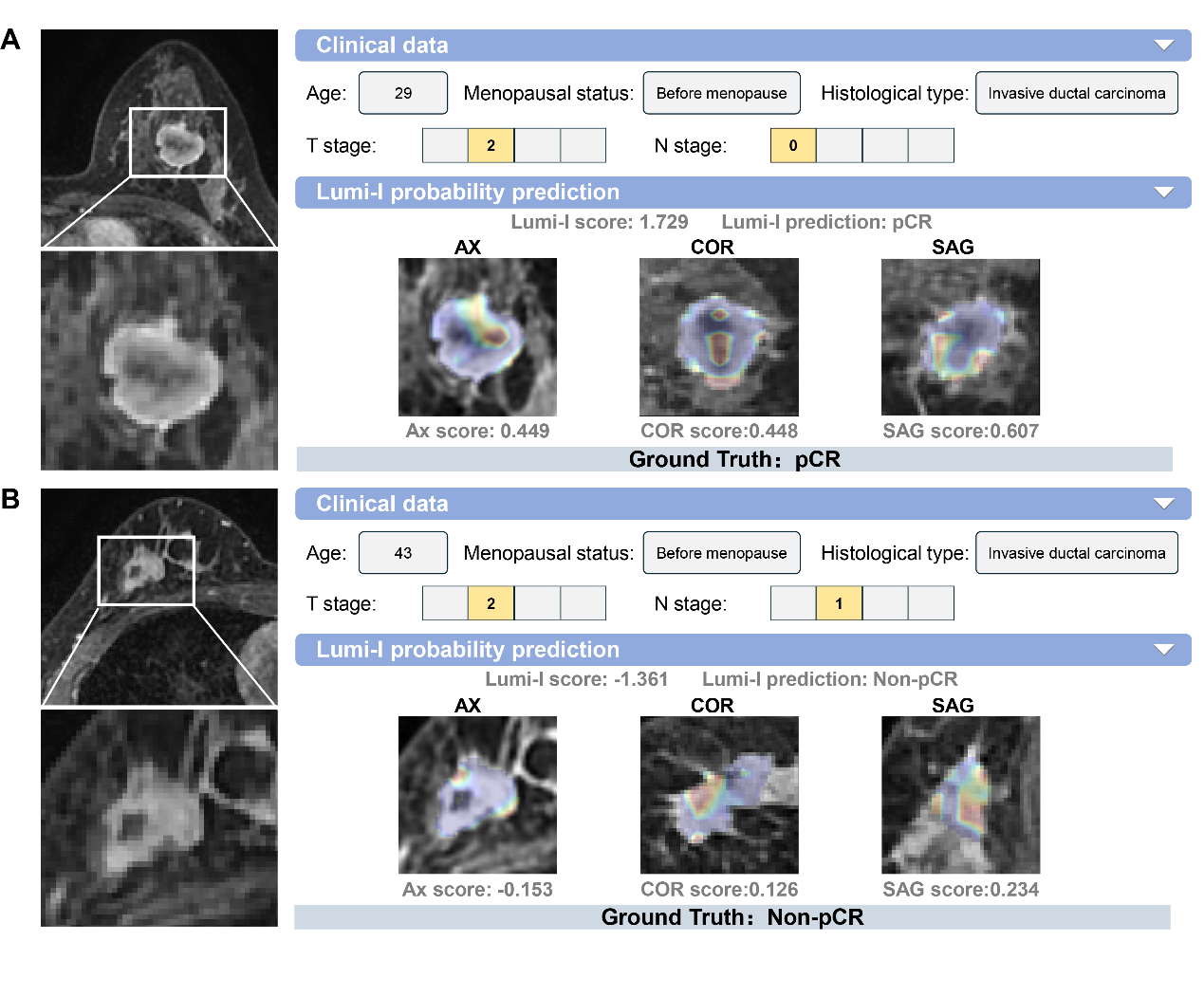


**Supplementary Fig. S2. Visualization of prediction results of the Lumi-I model.** Heatmap analysis of two representative cases. Red areas indicate high model attention, while blue areas indicate low model attention. **(A)** Example of a woman who achieved pCR after neoadjuvant therapy (NAT). The Lumi-I model correctly identified this patient as pCR. **(B)** Example of a woman who achieved non-pCR after NAT. The Lumi-I model correctly identified this patient as non-pCR. Abbreviations: pCR, pathological complete response; Lumi-I model, Luminal Breast Cancer Imaging model; AX, axial; COR, coronal; SAG, sagittal.


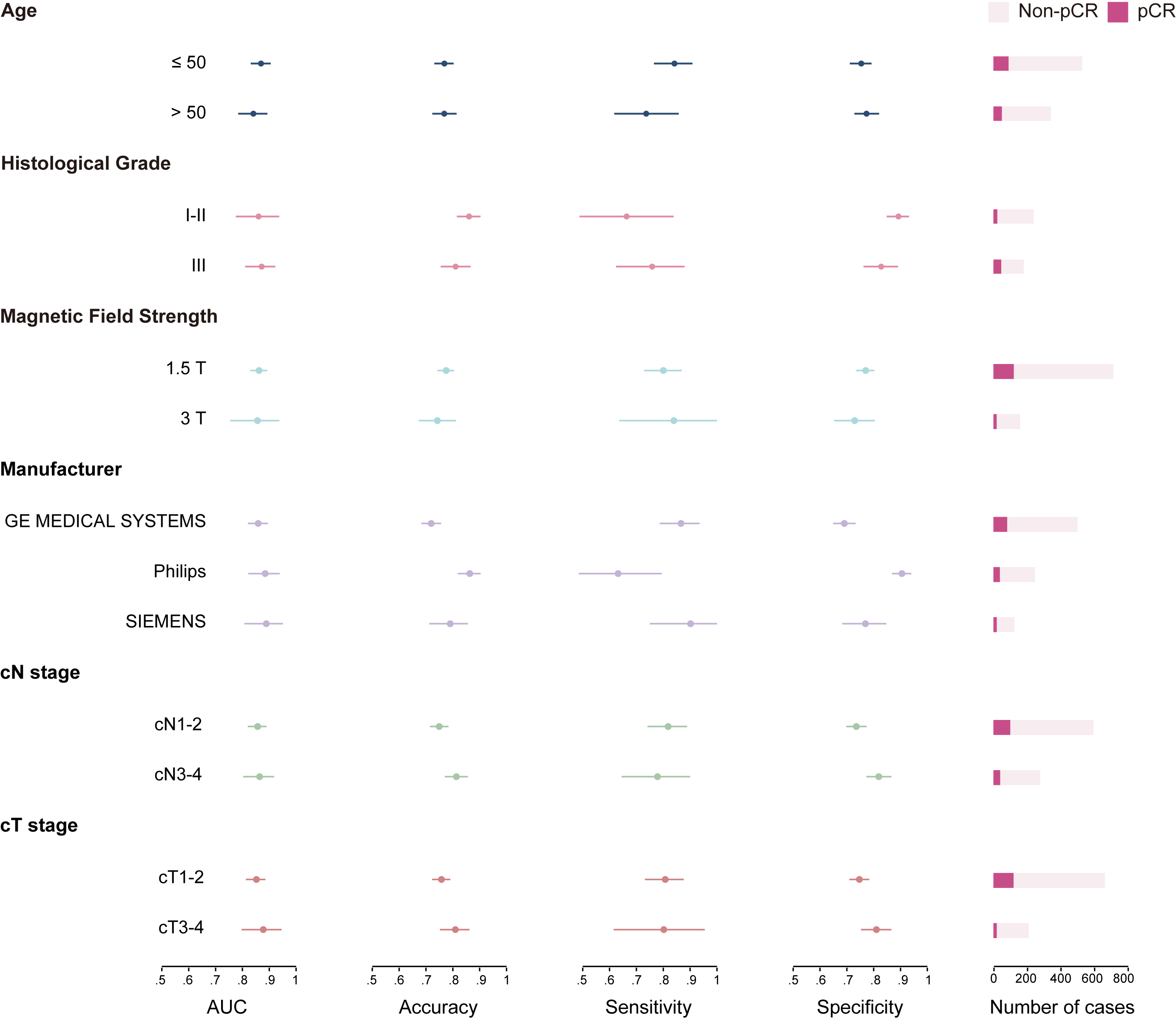


**Supplementary Fig. S3. Performance of Lumi-CI model on key subgroups on the combined training and validation sets.** Abbreviations: AUC, area under the curve.


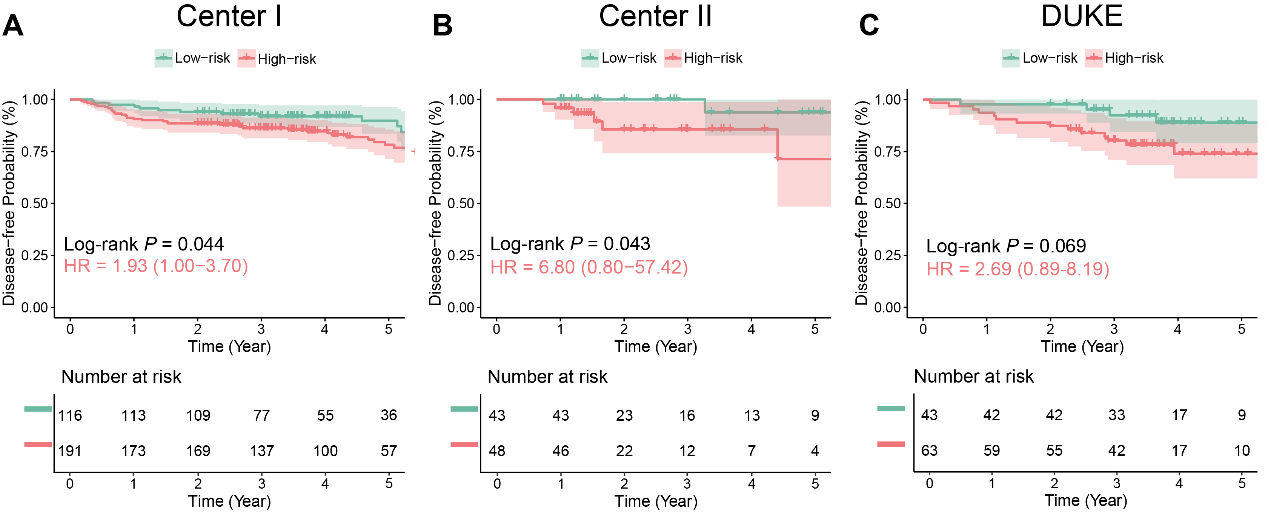


**Supplementary Fig. S4. Survival curves of risk groups stratified by the Lumi-CI model.** The Kaplan–Meier survival curves of DFS in **(A)** Center I, **(B)** Center II, and **(C)** DUKE.


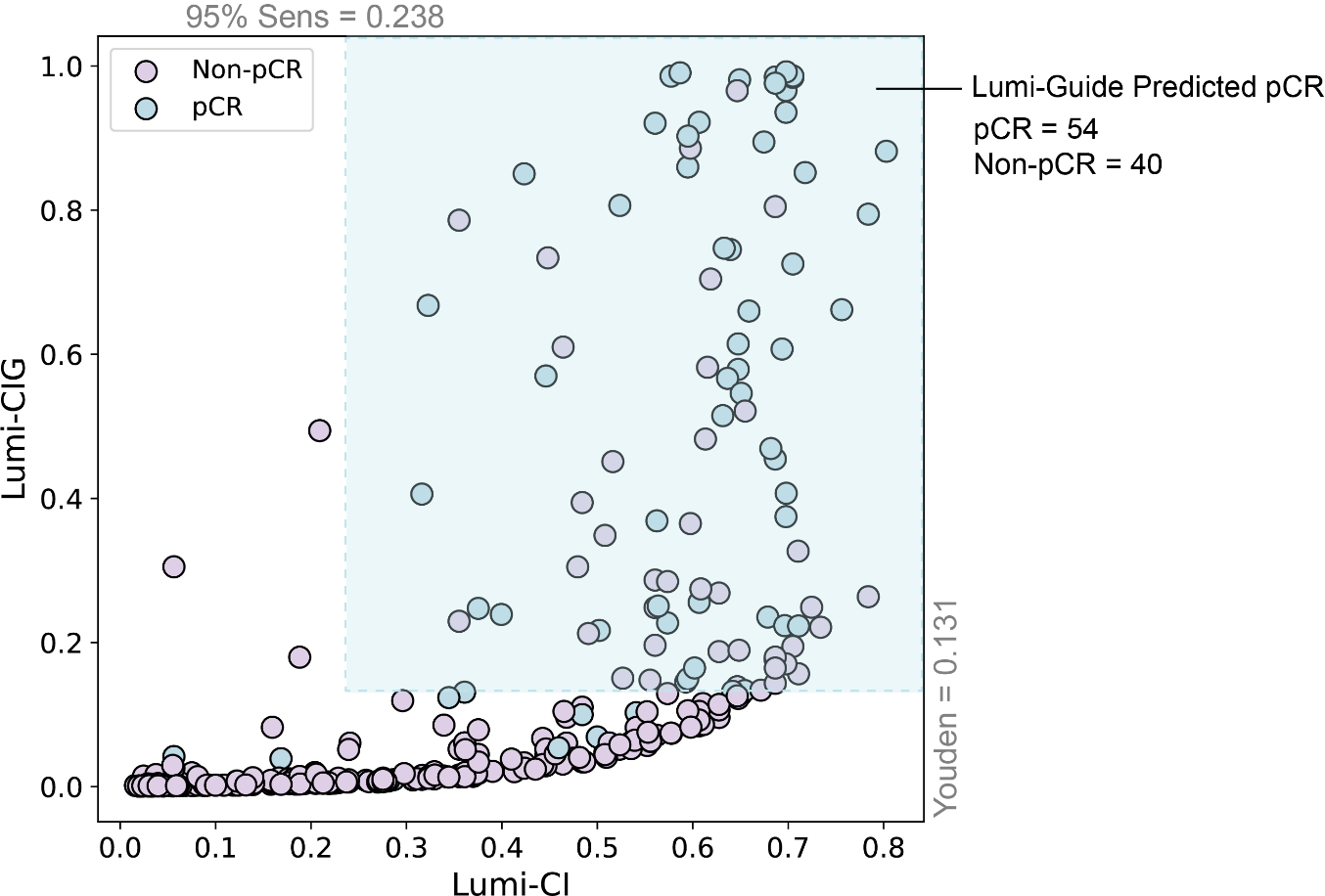


**Supplementary Fig. S5. Patient distribution and decision thresholds in the two-step triage Lumi-Guide system based on Lumi-CI and Lumi-CIG models.** The system applies a 95% sensitivity threshold (0.238) for the Lumi-CI model to exclude patients unlikely to achieve pCR, while the Lumi-CIG model uses Youden index-optimized threshold (0.131) for final pCR prediction. The highlighted region identifies patients predicted to achieve pathological complete response by Lumi-Guide system. Abbreviations: Lumi-CI model, Luminal Breast Cancer Clinical-Imaging model; Lumi-CIG model, Luminal Breast Cancer Clinical-Imaging-Genomic model; Lumi-Guide system, Luminal Breast Cancer Guidance system; Sens, sensitivity; pCR, pathological complete response.

**
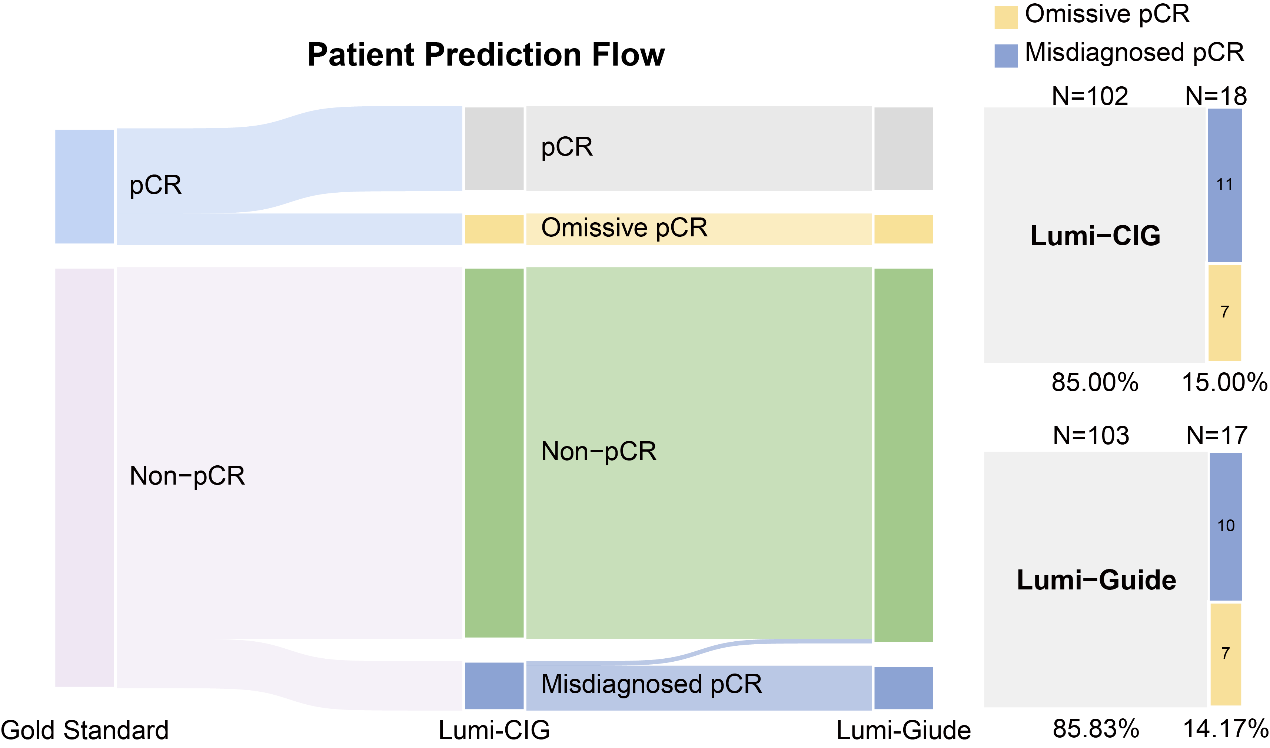
**

**Supplementary Fig. S6. Prediction results flow between Lumi-CIG and Lumi-Guide in the radiogenomics validation set.** Abbreviations: Lumi-CIG model, Luminal Breast Cancer Clinical-Imaging-Genomic model; Lumi-Guide system, Luminal Breast Cancer Guidance system; pCR, pathological complete response.


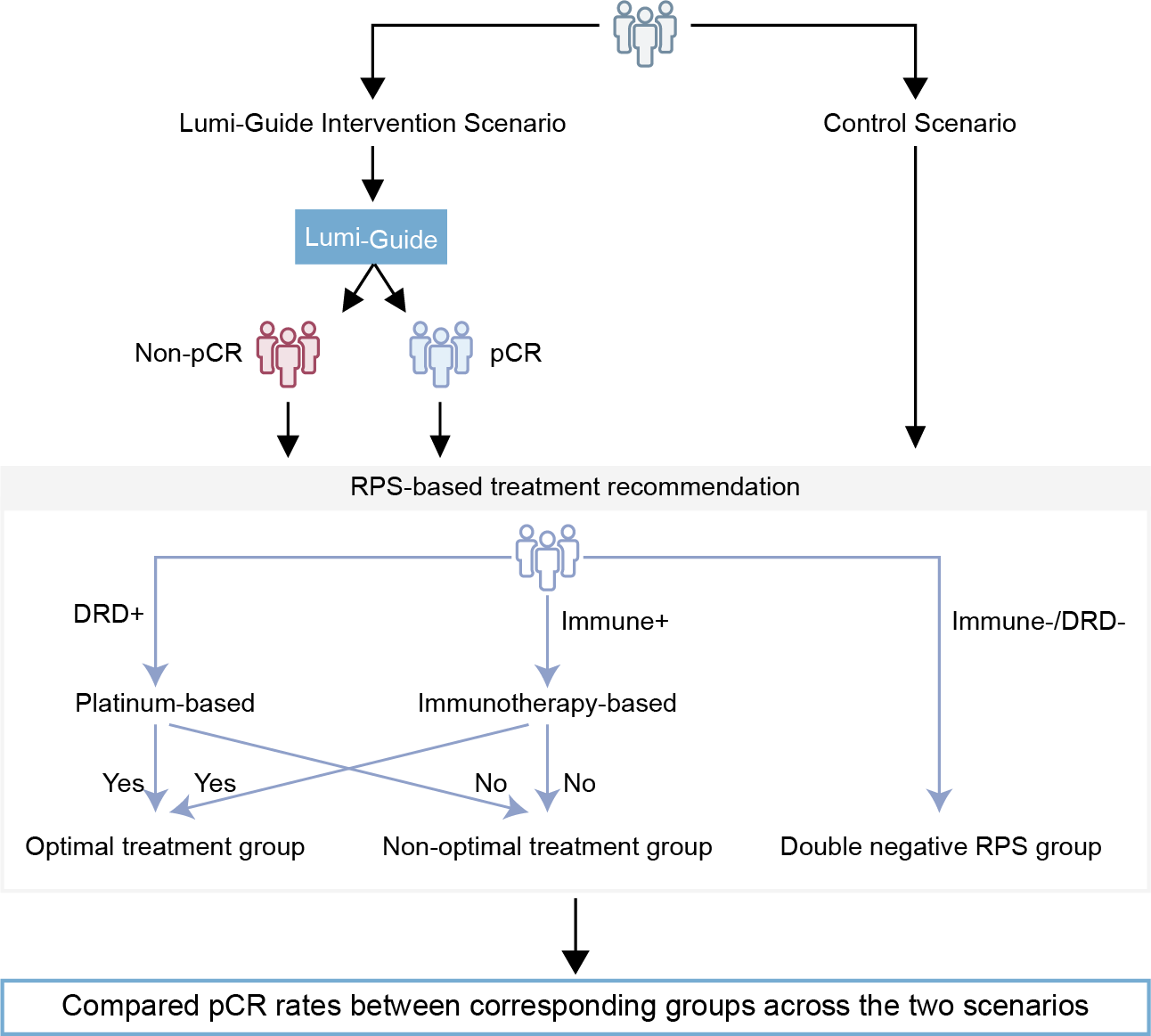


**Supplementary Fig. S7. Schematic of the counterfactual analysis that compare RPS-guided treatment with and without Lumi-Guide pre-stratification.** Abbreviations: RPS, response-predictive subtype; pCR, pathological complete response; Lumi-Guide system, Luminal Breast Cancer Guidance system.

**
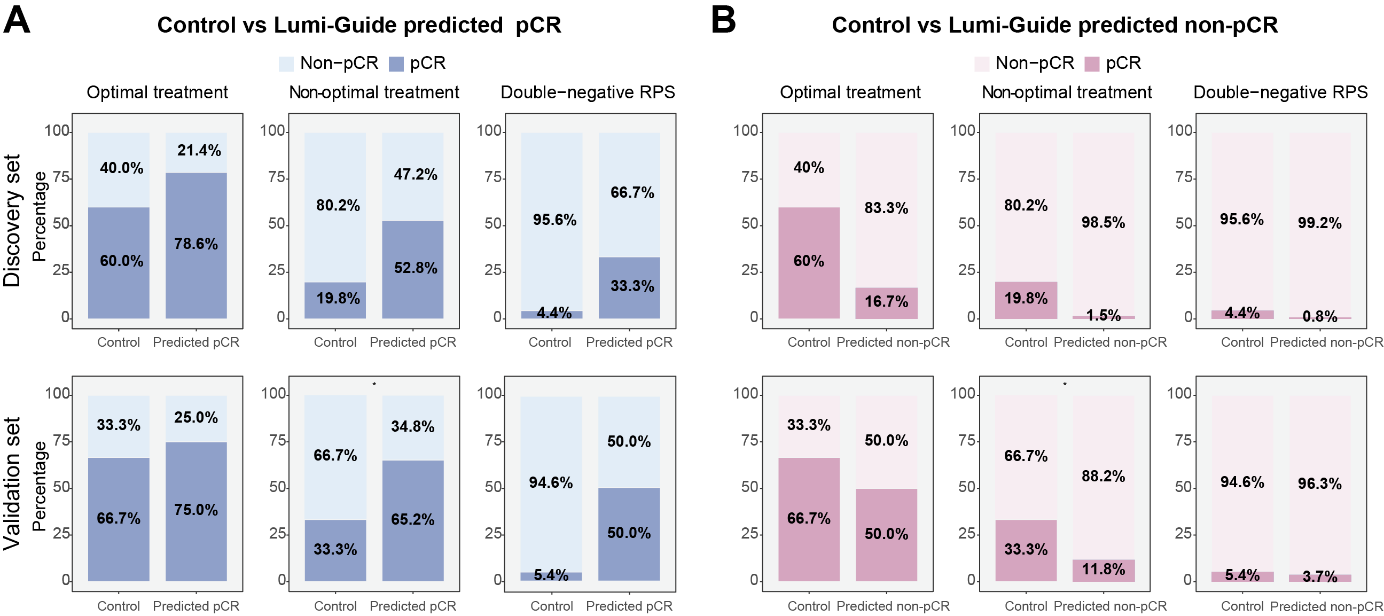
**

**Supplementary Fig. S8. Stratified simulation analysis of Lumi-Guide system pre-stratification performance across RPS-guided treatment patterns in radiogenomics discovery and validation sets. (A)** Pathological complete response rates in Lumi-Guide system predicted pCR patients compared to controls across optimal treatment, non-optimal treatment, and double-negative RPS groups in the radiogenomics discovery and validation sets. **(B)** Pathological complete response rates in Lumi-Guide system predicted non-pCR patients compared to controls across optimal treatment, non-optimal treatment, and double-negative RPS groups in the radiogenomics discovery and validation sets. Abbreviations: RPS, response-predictive subtype; pCR, pathological complete response; Lumi-Guide system, Luminal Breast Cancer Guidance system.


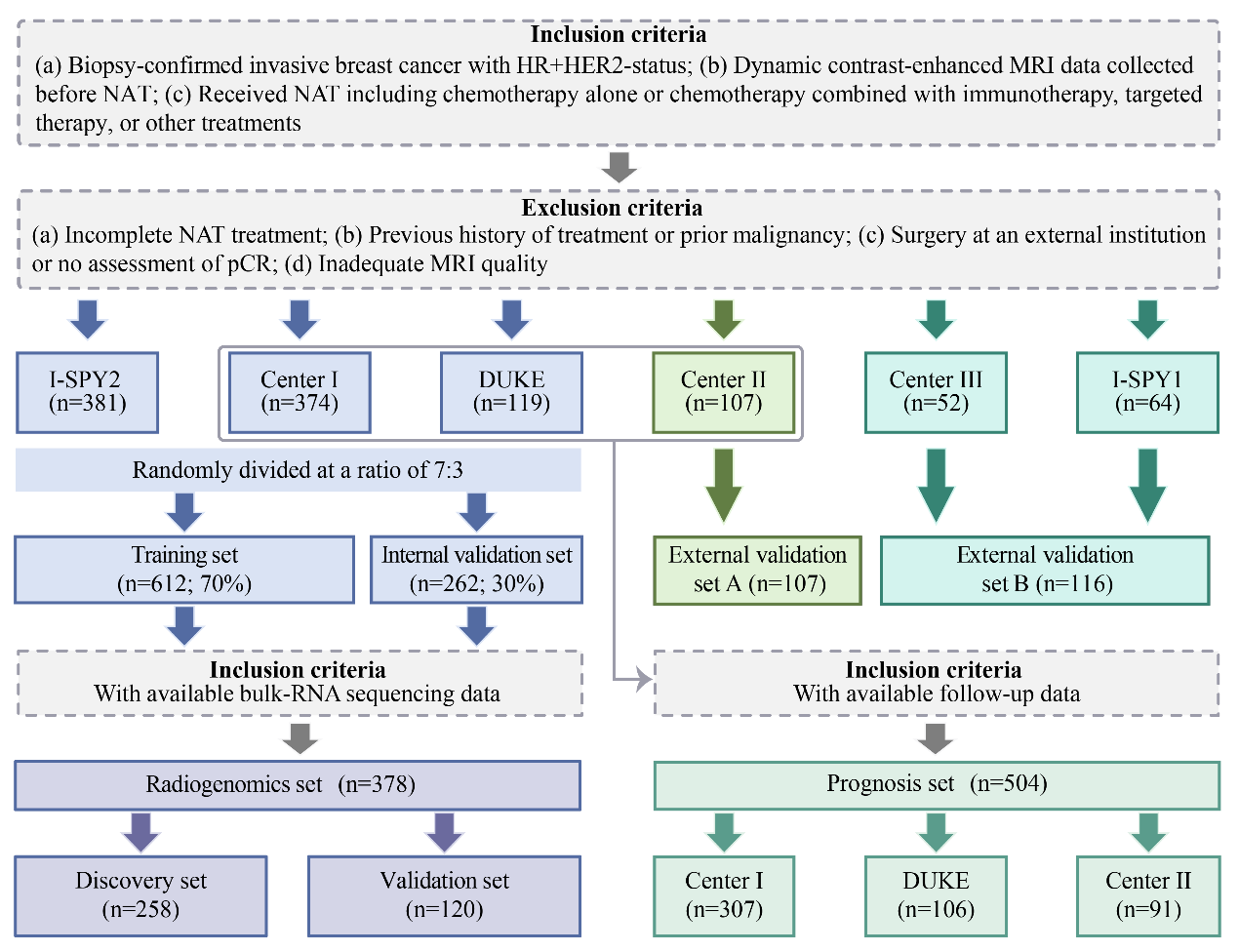


**Supplementary Fig. S9. Flowchart of the study datasets.** Following inclusion and exclusion criteria, a total of 1,097 patients from six datasets were included in this study. Patients from Center I, I-SPY2, and DUKE datasets (n=874) underwent stratified random allocation into training (70%) and internal validation (30%) sets, with radiogenomics discovery (n=258) and validation (n=120) sets derived from samples with available bulk-RNA sequencing data. External validation was performed using two independent test sets. The prognosis set was derived from patients with available follow-up data.

**Supplementary Table S1. Univariable and multivariable analysis of clinical factors potentially affecting pathological complete response.**

| **Characteristic** | **Univariable logistic analysis** | |  | **Multivariable logistic analysis** | |
| --- | --- | --- | --- | --- | --- |
|  | **OR** | ***P*** |  | **OR** | ***P*** |
| Age | 0.98 (0.97-0.99) | 0.003^*^ |  | 0.99 (0.98-1.01) | 0.281 |
| Pre-NAC clinical T stage |  |  |  |  |  |
| cT1 | Reference |  |  |  |  |
| cT2 | 0.86 (0.57-1.31) | 0.486 |  | 0.62 (0.38-1.01) | 0.054 |
| cT3 | 0.43 (0.25-0.72) | 0.002^*^ |  | 0.48 (0.26-0.89) | 0.020^*^ |
| cT4 | 0.48 (0.25-0.94) | 0.031^*^ |  | 0.55 (0.24-1.25) | 0.155 |
| Pre-NAC clinical N stage |  |  |  |  |  |
| cN0 | Reference |  |  |  |  |
| cN1 | 0.55 (0.41-0.73) | <0.001^*^ |  | 0.61 (0.43-0.87) | 0.007^*^ |
| cN2 | 0.54 (0.38-0.77) | <0.001^*^ |  | 0.55 (0.36-0.85) | 0.007^*^ |
| cN3 | 0.51 (0.29-0.90) | 0.020^*^ |  | 0.85 (0.42-1.72) | 0.658 |

Note: ^*^ Indicates statistical significance: *P* < 0.05. Abbreviations: OR, odds ratio.

**Supplementary Table S2. The performance of models in predicting pathological complete response**

| **Datasets** | **Models** | **AUC**  **(95% CI)** | **Accuracy**  **(95% CI)** | **Sensitivity**  **(95% CI)** | **Specificity**  **(95% CI)** |
| --- | --- | --- | --- | --- | --- |
| Training | Lumi-C model | 0.610  (0.543-0.671) | 0.680  (0.641-0.717) | 0.469  (0.367-0.565) | 0.720  (0.678-0.760) |
|  | Lumi-I model | 0.822  (0.781-0.859) | 0.691  (0.654-0.729) | 0.857  (0.778-0.919) | 0.660  (0.616-0.698) |
|  | Lumi-CI model | 0.883  (0.848-0.912) | 0.775  (0.739-0.806) | 0.857  (0.779-0.921) | 0.759  (0.719-0.794) |
| Validation | Lumi-C model | 0.558  (0.480-0.650) | 0.691  (0.641-0.748) | 0.381  (0.235-0.528) | 0.750  (0.696-0.806) |
|  | Lumi-I model | 0.799  (0.734-0.869) | 0.698  (0.649-0.752) | 0.762  (0.635-0.884) | 0.686  (0.628-0.748) |
|  | Lumi-CI model | 0.810  (0.741-0.880) | 0.760  (0.710-0.809) | 0.690  (0.558-0.824) | 0.773  (0.717-0.827) |
| External test set A | Lumi-C model | 0.606  (0.442-0.766) | 0.673  (0.579-0.757) | 0.429  (0.166-0.688) | 0.710  (0.614-0.802) |
|  | Lumi-I model | 0.803  (0.664-0.922) | 0.729  (0.645-0.813) | 0.643  (0.357-0.889) | 0.742  (0.652-0.830) |
|  | Lumi-CI model | 0.819  (0.715-0.908) | 0.804  (0.720-0.879) | 0.571  (0.286-0.833) | 0.839  (0.758-0.915) |
| External test set B | Lumi-C model | 0.613  (0.410-0.797) | 0.647  (0.552-0.733) | 0.500  (0.222-0.786) | 0.663  (0.566-0.755) |
|  | Lumi-I model | 0.802  (0.663-0.925) | 0.698  (0.612-0.776) | 0.750  (0.467-1.000) | 0.692  (0.604-0.777) |
|  | Lumi-CI model | 0.864  (0.751-0.947) | 0.802  (0.724-0.871) | 0.667  (0.385-0.923) | 0.817  (0.738-0.887) |

Abbreviations: AUC, area under the curve; CI, confidence interval; Lumi-C model, Luminal Breast Cancer Clinical model; Lumi-I model, Luminal Breast Cancer Imaging model; Lumi-CI model, Luminal Breast Cancer Clinical-Imaging model.

**Supplementary Table S3. The detailed calculation method of each biomarker.**

| **Biomarker** | **Genes** | **PMID** |
| --- | --- | --- |
| Module5 Tcell-Bcell score | IGSF6, LILRB2, BTN3A3, UBD, CXCL13, GNLY, CXCR6, CTSC, HCP5, PIM2, SP140, CCR7, CTSS, CYBB, FCN1, TFEC, SEL1L3, FYB, GBP1, LAMP3, ADAMDEC1, GPR18, ICOS, GPR171, GZMH, GZMB, GZMK, BIRC3, IFNG, IL2RG, IL15, IDO1, CXCL10, IRF1, ISG20, ITK, LAG3, LCK, LYN, CXCL9, NKG7, TRAT1, MGC29506, PLAC8, POU2AF1, CRTAM, SLAMF8, PSMB9, PTPN7, SLAMF7, BCL2A1, TNFRSF17, CCL5, CCL8, CCL13, CCL18, CCL19, CXCL11, SELL, SAMSN1, RTP4, CLEC7A, TAP1, WARS, PLA2G7, ZBED2, NPL, RUNX3, VNN2, CD3G, IL32, CD8B, CD19, CD86, AIM2, CD38, CYTIP, LOC96610, CD69, CD79A | 24516633 |
| ICS5 score | CXCL13, CLIC5, HLA-F, TNFRSF17, XCL2 | 24172169 |
| B cells | BLK, CD19, FCRL2, KIAA0125, MS4A1, PNOC, SPIB, TCL1A, TNFRSF17 | 28239471 |
| Dendritic cells | CCL13, CD209, HSD11B1 | 28239471 |
| Mast cells | CPA3, HDC, MS4A2, TPSAB1, TPSB2 | 28239471 |
| STAT1 sig | TAP1, GBP1, IFIH1, PSMB9, CXCL9, IRF1, CXCL11, CXCL10, IDO1, STAT1 |  |
| Chemokine12 score | CCL2, CCL3, CCL4, CCL5, CCL8, CCL18, CCL19, CCL21, CXCL9, CXCL10, CXCL11, CXCL13 | 21703392 |
| Module11 Prolif score | CDKN3, NDC80, RNASEH2A, CENPA, SMC2, CENPE, RAD51AP1, PLK4, NMU, KIF2C, TMSB15A, UBE2C, CHEK1, ZWINT, OIP5, CRABP1, ECT2, EIF4EBP1, EZH2, FEN1, HSPA4L, TPX2, FOXM1, NCAPH, PRAME, PDSS1, KIF4A, RAD54B, ASPM, FBXO5, ATAD2, RACGAP1, GPSM2, DONSON, HMMR, BIRC5, KIF11, LMNB1, MAD2L1, MCM4, MCM5, MKI67, MMP1, MYBL1, MYBL2, NEK2, NUSAP1, GTSE1, GINS2, PLK1, FAM64A, ERCC6L, NCAPG2, CEP55, FANCI, HJURP, MCM10, DEPDC1, C1orf112, CENPN, PBK, KIF15, CIAPIN1, ACTR3B, GPR126, SPC25, RAD21, RFC3, RFC4, RRM2, NCAPG, STIL, SKP2, SOX11, SQLE, AURKA, TAF2, TARS, BUB1B, TK1, TMPO, TOP2A, PHLDA2, TTK, LRP8, DSCC1, MLF1IP, E2F8, SHCBP1, SLC7A5, ANP32E, KIF18A, CDC7, CDC45, RAD54L, TTF2, PIR, ACTL6A, GGH, CCNA2, CCNB1, PRC1, CCNB2, CCNE2, EXO1, AURKB, PTTG1, TRIP13, KIF23, APOBEC3B, MTFR1, ESPL1, DLGAP5, CDK1, MELK, GINS1, CDC6, CDC20, NCAPD2, KIF14 | 24516633 |
| MP index | AA834945, AI224578, AI283268, ALDH4A1, AP2B1, AW014921, AYTL2, BBC3, C16orf61, C20orf46, C9orf30, CDC42BPA, CDCA7, CENPA, COL4A2, DCK, DIAPH3, DIAPH3, DIAPH3, DKFZP686P18101, DTL, ECT2, EGLN1, ESM1, EXT1, FBXO31, FGF18, FLT1, GMPS, GNAZ, GPR126, GPR180, GSTM3, HRASLS, IGFBP5, IGFBP5, KNTC2, LGP2, LOC286052, LOC643008, MCM6, MELK, MMP9, MS4A7, MTDH, NMU, NM_004702, NUSAP1, ORC6L, OXCT1, PALM2-AKAP2, PECI, PECI, PITRM1, PQLC2, PRC1, QSCN6L1, RAB6A, RFC4, RP5-860F19.3, RTN4RL1, RUNDC1, SCUBE2, SLC2A14, STK32B, TGFB3, TSPYL5, UCHL5, WISP1, ZNF533 | 11823860 |
| Basal Index | ABCC11, ACADSB, AFF3, AGF2, AR, CA12, CAPN13, CDCA7, CHAD, DHRS2, ESR1, FOXA1, FOXC1, GATA3, GREB1, KIAA1370, MAGED2, MLPH, MSN, MYO5C, PERLD1, PRR15, REEP6, RTN4L1, SLC16A6, SPEF1, TBC1D9, THSD4 | 21814749 |
| ER PGR avg | ESR1, PGR | 35623341 |
| Luminal Index | ABAT, ACADSB, ACBD4, ADM, AFF3, BCL2, BECN1, BTD, BTRC, CA12, CCDC74B, CDC25B, CELSR1, CELSR2, CHAD, COQ7, DNALI1, ELOVL5, ESR1, GATA3, GOLSYN, GREB1, HDAC11, HK3, HMGCL, IL6ST, IRS1, KIAA1737, KIF20A, LILRB3, LRIG1, MYB, NAT1, NPY1R, NUDT6, OCIAD1, PARD6B, PGR, PPAPDC2, PREX1, RERG, RUNDC1, S100A8, SCUBE2, SOX11, SUSD3, TAPT1, TBC1D9, TCTN1, THSD4, TMC4, TMEM101, TMSB10, TPRG1, UBXD3, DBNDD2, VAV3, XBP1 | 21814749 |
| PARPi7 score | Prediction genes: BRCA1, CHEK2, MAPKAPK2, MRE11A, NBN, TDG, XPA; Normalization genes: RPL24, ABI2, GGA1, E2F4, IPO8, CXXC1, RPS10 | 28948212 |
| VCpred TN | CXCL13, BRCA1, APEX1, FEN1, CD8A, SEM1 (SHFM1), APEX2, RNMT, CCR7, H2AFX, POLD3, PRKDC, C1QA, CLIC5, RAD51, DDB2, SPP1, POLD2, POLB, LIG1, GTF2H5, PMS2, LY9, SHPRH, ARAF | 35623341 |
| IGF1R dat | IGF1R | 33083527 |
| STMN1 dat | STMN1 | 32914002 |
| LYMPHS PCA | UQCRB, SESTD1, QTRT1, TIPIN, REL, STXBP2, HSBP1, COX6C, RPL11, MECOM, ANKRD28, JUN, ZC3H15, RPL23, RPS6KA2, EEF2, TMA7, RPS6, RPL27, RPS21, COX7B, PRRC2B, CYP17A1, NSUN4, TOMM34, MINOS1, STAMBPL1, FGF9, ATF4, RPL35, RPL31, RPS24, DCLRE1C, C5orf49, FAM162A, ITGB2, SLC19A1, RPL32, TPP2, MALAT1, LSM3, TSSC1, ATXN2L, SERPINB6, TPI1 | 16704732 |
| Module7 ERBB2 score | ERBB2, GRB7, STARD3, PGAP3 | 24516633 |
| PAM50 subtype | ACTR3B, ANLN, BAG1, BCL2, BIRC5, BLVRA, CCNB1, CCNE1, CDC20, CDC6, CDCA1, CDH3, CENPF, CEP55, CXXC5, EGFR, ERBB2, ESR1, EXO1, FGFR4, FOXA1, FOXC1, GPR160, GRB7, KIF2C, KNTC2, KRT14, KRT17, KRT5, MAPT, MDM2, MELK, MIA, MKI67, MLPH, MMP11, MYBL2, MYC, NAT1, ORC6L, PGR, PHGDH, PTTG1, RRM2, SFRP1, SLC39A6, TMEM45B, TYMS, UBE2C, UBE2T | 19204204 |

**Supplementary Table S4. The performance of Lumi-CI, Lumi-G, Lumi-CIG models and Lumi-Guide system in predicting pCR.**

| **Datasets** | **Models** | **AUC**  **(95% CI)** | **Accuracy**  **(95% CI)** | **Sensitivity**  **(95% CI)** | **Specificity**  **(95% CI)** | **F1 score**  **(95% CI)** | **Precision**  **(95% CI)** |
| --- | --- | --- | --- | --- | --- | --- | --- |
| The radiogenomics discovery set | Lumi-G model | 0.911  (0.859-0.956) | 0.880  (0.841-0.919) | 0.816  (0.692-0.933) | 0.891  (0.850-0.932) | 0.667  (0.543-0.776) | 0.564  (0.432-0.694) |
|  | Lumi-CI model | 0.875  (0.818-0.924) | 0.667  (0.609-0.721) | 0.947  (0.875-1.000) | 0.618  (0.554-0.679) | 0.456  (0.355-0.543) | 0.300  (0.222-0.381) |
|  | Lumi-CIG model | 0.955  (0.924-0.980) | 0.864  (0.822-0.903) | 0.921  (0.828-1.000) | 0.855  (0.806-0.900) | 0.667  (0.554-0.762) | 0.522  (0.403-0.639) |
|  | Lumi-Guide system | 0.892  (0.840-0.939) | 0.872  (0.829-0.911) | 0.921  (0.828-1.000) | 0.864  (0.815-0.907) | 0.680  (0.568-0.776) | 0.538  (0.421-0.661) |
| The radiogenomics validation set | Lumi-G model | 0.825  (0.751-0.888) | 0.742  (0.667-0.817) | 0.462  (0.267-0.650) | 0.819  (0.739-0.890) | 0.436  (0.255-0.592) | 0.414  (0.238-0.594) |
|  | Lumi-CI model | 0.822  (0.736-0.904) | 0.692  (0.608-0.775) | 0.846  (0.697-0.963) | 0.649  (0.557-0.747) | 0.543  (0.400-0.660) | 0.400  (0.273-0.525) |
|  | Lumi-CIG model | 0.859  (0.778-0.931) | 0.850  (0.783-0.908) | 0.731  (0.565-0.895) | 0.883  (0.816-0.944) | 0.679  (0.524-0.806) | 0.633  (0.448-0.800) |
|  | Lumi-Guide system | 0.812  (0.726-0.899) | 0.858  (0.800-0.917) | 0.731  (0.565-0.895) | 0.894  (0.830-0.956) | 0.691  (0.538-0.818) | 0.655  (0.474-0.833) |

Abbreviations: pCR, pathological complete response; AUC, area under the curve; CI, confidence interval; Lumi-CI model, Luminal Breast Cancer Clinical-Imaging model; Lumi-G model, Luminal Breast Cancer Genomic model; Lumi-CIG model, Luminal Breast Cancer Clinical-Imaging-Genomic model; Lumi-Guide system, Luminal Breast Cancer Guidance system.

**Supplementary Table S5. Imaging acquisition parameters across different centers.**

| **Centers** | **Scanner** | **Field strength** | **Sequence** | **Slice thickness (mm)** | **FOV**  **(mm)** | **Echo time**  **(ms)** | **Repetition time**  **(ms)** |
| --- | --- | --- | --- | --- | --- | --- | --- |
| Center I | GE, Philips | 1.5T, 3T | T1+C | 1.0-2.0 | 319-420 | 2.27-2.66 | 5.03-5.74 |
| Center II | GE, SIEMENS | 1.5T, 3T | T1+C | 1.0-1.1 | 288-384 | 1.67-1.69 | 4.80-5.30 |
| Center III | United Imaging | 1.5 T | T1+C | 1.2 | 320-320 | 2.1 | 5.1 |
| DUKE | GE, SIEMENS | 1.5T, 3T | T1+C | 1.04-2.5 | 270-440 | 1.27-2.704 | 3.54-5.13 |
| I-SPY1 | GE, SIEMENS, Philips | 1.5 T | T1+C | 1 | 160–180 | 4.5 | ≤ 20 |
| I-SPY2 | GE, SIEMENS, Philips | 1.5T, 3T | T1+C | 0.9-2.5 | 260–360 | 1.37-4.78 | 4.00-9.06 |

Abbreviations: T1+C, contrast-enhanced T1-weighted imaging; mm, millimeter; ms, millisecond.

**Supplementary Table S6. Optimal** **hyperparameters for the Lumi-CI and Lumi-G models.**

| **Model** | **Estimator** | **Tree depth** | **Subsample** | **Colsample** |
| --- | --- | --- | --- | --- |
| Lumi-CI model | 18 | 2 | 0.4 | 0.8 |
| Lumi-G model | 24 | 2 | 0.4 | 0.6 |

Abbreviations: Lumi-CI model, Luminal Breast Cancer Clinical-Imaging model; Lumi-G model, Luminal Breast Cancer Genomic model.

**Supplementary Table S7. Radiomic features retained for model construction.**

| **Number** | **Filter type** | **Feature type** | **Feature name** | ***P* value** |
| --- | --- | --- | --- | --- |
| 1 | Original | Shape | Sphericity | <0.001 |
| 2 | Square | First order | Energy | 0.007 |
| 3 | Square | First order | Maximum | 0.039 |
| 4 | Square | GLCM | MaximumProbability | 0.034 |
| 5 | Wavelet.LHL | First order | Skewness | 0.035 |
| 6 | Wavelet.LHL | GLCM | MCC | 0.019 |
| 7 | Wavelet.LHH | First order | Median | 0.005 |
| 8 | Wavelet.HLL | First order | Skewness | 0.002 |
| 9 | Wavelet.HLL | GLCM | Imc1 | 0.02 |
| 10 | Wavelet.HHL | First order | Median | 0.033 |
| 11 | Wavelet.LLL | GLDM | LargeDependenceLowGrayLevelEmphasis | 0.019 |
| 12 | Wavelet.LLL | GLRLM | ShortRunLowGrayLevelEmphasis | 0.006 |
